# Hierarchical Summary Statistics Encoding Across Primary Visual and Posterior Parietal Cortices

**DOI:** 10.1002/advs.202512369

**Published:** 2026-03-22

**Authors:** Young‐Beom Lee, Oliver James, Gaeun Jung, Doyun Lee, Yee‐Joon Kim

**Affiliations:** ^1^ Center For Memory and Glioscience Institute For Basic Science Daejeon South Korea; ^2^ Department of Psychology Chungnam National University Daejeon South Korea

**Keywords:** distributed population code, ensemble perception, perceptual categorization, summary statistics encoding, visual cortical hierarchy

## Abstract

Despite growing evidence that the visual system pools sensory data into a summary statistical representation, the underlying neural mechanisms remain unclear. We characterized the neural coding of summary statistics at the single‐cell and population levels using calcium signals imaged in primary visual cortex (V1) and posterior parietal cortex (PPC) while head‐fixed mice passively viewed or classified eight mean motion directions of randomly moving dots into two categories. A small portion of neurons in both areas showed global mean motion direction selectivity beyond what would be expected from the simple summation of responses to individual dot motions. Although this selectivity was variable across stimulus variability and trials, population activity robustly encoded global mean motion direction, even though most neurons were not significantly tuned. The V1 population‐level mean motion representation was dependent on stimulus variance and systematically biased toward the category center during the motion categorization task. These, along with the observed population‐level neural coding of stimulus variance, suggest that multivariate V1 activity is well suited to processing summary statistics. The redundant summary statistical encodings in both V1 and PPC suggest that such information accumulates across the visual hierarchy, which may allow PPC to bind multiple levels of summary statistical representations into task‐oriented category signals.

## Graphical abstract

This study shows that mouse V1 simultaneously encodes the ensemble mean and variance of motion, providing a robust summary‐statistic representation that persists despite single‐neuron variability. These signals propagate to PPC, where they are transformed into abstract category representations during decision making. The findings reveal a hierarchical compression of sensory detail into increasingly abstract statistical codes.

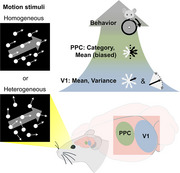

## 1 Introduction

In order for animals to effectively navigate complex visual environments, the visual system is required to rapidly distill the vast amount of sensory data into a compact abstract representation that captures important image structure [1, 2]. One parsimonious mechanism is to exploit the statistical dependencies found in real‐world scenes to represent a large amount of visual information, often extending into the visual periphery, as summary statistics such as mean and variance or range) [3, 4, 5, 6, 7]. Extracting stable information, such as sensory feature averages from dynamic sensory environments, helps optimize behaviors by allowing the structure of the environment to be robustly grasped. This ensemble perception has been suggested to evoke our subjective impression of a rich visual world despite the irretrievable loss of the detailed visual information [8, 9, 10]. Due to the ubiquitous nature of statistical representations in perception, it has been suggested that they may serve as the basis for rapid visual categorization [11, 12, 13, 14, 15, 16, 17], another scene organizing mechanism for facilitating perception and cognition. Summary statistics, as mechanisms for helping to overcome perceptual bottlenecks and perceive the gist of the scene [9, 14, 16, 18], have been extensively studied. Yet, it remains unclear how the visual system processes concrete sensory information into increasingly abstract information such as the mean feature value along the cortical hierarchy.

While recent computational models have proposed that pooled population responses in hierarchically organized networks provide a perceptually compressive representation of summary statistics [19, 20, 21, 22, 23], it is not known exactly at what level of the visual cortical hierarchy summary statistical information is represented. In the case of motion processing, one monkey study has suggested that the medial temporal (MT) area pools local motion vector signals from V1 into global mean motion encoding even though this direction is physically absent in the stimulus [24]. Computationally, the pooling stage implements a weighted average over feature‐selective channels. In the motion domain, when local directions are sampled from a distribution centered on a mean, the pooled population response forms a smooth profile whose peak coincides with the circular mean, and whose width scales with dispersion. Thus, the ensemble mean can emerge as a natural consequence of feedforward integration, without requiring explicit item‐wise estimation. However, because similar pooling operations recur across the visual hierarchy, summary statistics such as mean and variance could, in principle, arise at multiple stages [25], making it difficult to determine where and how these statistics are encoded. To pinpoint the neural loci and mechanisms involved in coding summary statistics, it is necessary to use a task paradigm that can manipulate mean and variance independently, thereby isolating their respective contributions to neural responses.

To address this question, we used an unconventional random dot kinematogram (RDK), where the motion direction of each dot was independently chosen from a uniform distribution, allowing us to systematically vary the range of the distribution while preserving the global motion direction along the mean [26]. We then recorded calcium signals from mouse V1 [27] and PPC [28, 29, 30, 31] while head‐fixed mice passively viewed RDKs or grouped eight mean motion directions of RDKs into two categories that were separated by a learned category boundary [28]. We found redundant encoding of global mean motion direction at the single‐cell and population level in both V1 and PPC regardless of whether mice performed the motion categorization task. We also found that multivariate activities additionally encoded the motion direction variance and the motion category in V1 and PPC, respectively.

## 2 Results

**FIGURE 1:**
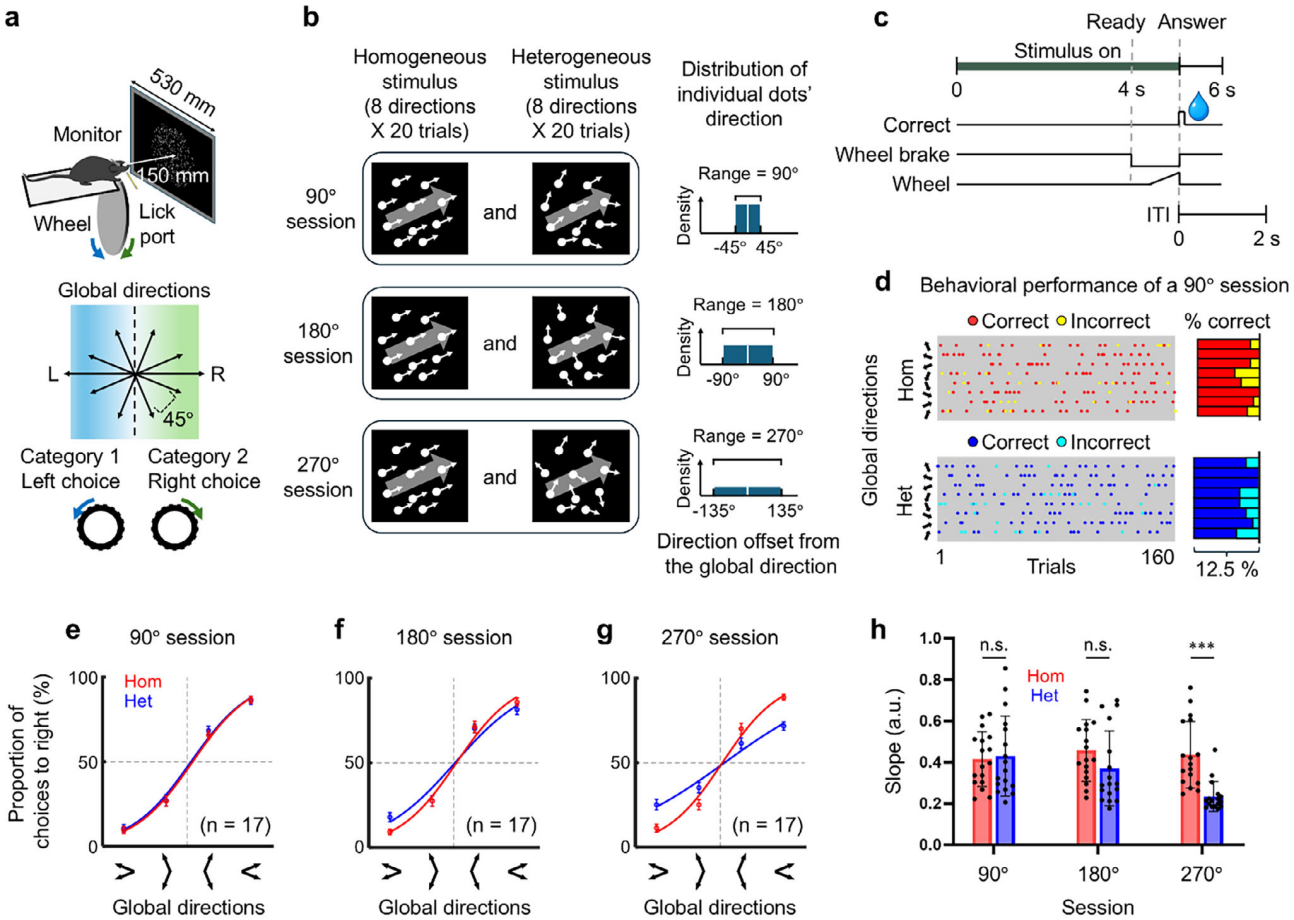
Experimental design, stimuli, trial sequence, and behavioral data analysis. (a) Schematic of the 2‐alternative forced choice motion categorization task. The head‐fixed mice were trained to group eight global motion directions into the two discrete categories by turning the wheel to the left or right. We used eight global motion directions from the predefined set of directions from 22.5° to 337.5° in steps of 45°. (b) Illustrations of how stimuli and conditions were created. First, one motion direction was sampled from eight possible motion directions and then used as a mean motion direction in each trial of the two conditions. In homogeneous random dot kinematograms (RDKs), the sampled motion direction was identical to the motion direction of the rest of the dots. In heterogeneous RDKs, each dot's motion direction was randomly selected from a uniform distribution within one of the three ranges (90°, 180°, and 270°) with one of eight global mean motion directions. Note that not a single dot in the heterogeneous RDK was assigned to the global motion direction along the distribution's mean. (c) Trial sequence of the motion categorization task. Mice viewed homogeneous or heterogeneous RDKs for 4 s. Wheel brake was released 4 s after the stimulus onset, and mice were provided with a maximum 2‐second response window to report their choice of motion category by turning a wheel either to the left or right. Correct responses were rewarded with a drop of water. The inter‐trial interval was 2 s. (d) Behavioral data in representative 90° test session. (left) The presented motion directions and categorical choices across homogeneous RDK trials (top) and heterogeneous RDK trials (bottom). (right) Performance correct for homogeneous (top) and heterogeneous conditions (bottom). See Table S1 for statistics. (e–g) Behavioral performance in three test sessions of 90° (e), 180° (f), and 270° (g). In each of three test sessions, the proportion of trials in which mice judged the global mean motion direction to belong to the “right” motion direction category for homogeneous (red) and heterogeneous (blue) conditions, respectively. Error bars indicate ±SEM. (h) The slope values of cumulative normal functions fit to individual mouse's behavioral data for homogeneous and heterogeneous conditions (e–g), respectively. Error bars indicate ±SEM. Data presented as the mean ± SEM, *N* = 17, *p*‐values were calculated using repeated one‐way ANOVA with Tukey's multiple comparisons test, ***p < 0.001.

To probe how well mice exploit and learn statistical regularities in visual scenes, we trained 17 head‐fixed mice to group eight global motion directions of RDK stimuli into two discrete categories separated by a learned vertical category boundary (Figure 1a). The heterogeneous RDK consisted of 500 dots moving in directions randomly drawn from a uniform distribution within a specific range centered around the distribution's mean. We used one of three different ranges across three test sessions conducted on different days: 90°, 180°, or 270° (Figure 1b; Movies S1–S3). Note that not a single dot in the heterogeneous RDK was assigned to the global motion direction corresponding to the distribution's mean. In each trial, we presented either homogeneous (Hom, a total of 160 trials) or heterogeneous RDKs (Het, a total of 160 trials) moving in one of eight mean directions equally spaced from 22.5° to 337.5° at a step of 45° (20 trials for each of the eight mean motion directions). The trial sequence within each session was pseudo‐randomized to prevent consecutive presentations of RDKs with the same global motion direction and the same motion variance. For 4 s after the stimulus onset, the decision wheel was locked by horizontally pressing a rubber‐padded linear actuator against the surface of the rubber‐coated wheel, thereby preventing rotation. After this period, the brake was released, and water‐restricted mice were provided with a 2‐second response window to report their categorical decision by turning the wheel either to the left or right. Correct responses were rewarded with a drop of water (Figure 1c). Figure 1d shows the behavioral performance across trials in one representative 90° test session. In each category of homogeneous (the top left in Figure 1d) and heterogeneous (the bottom left in Figure 1d) trials, behavioral performance was higher on trials with ‘easy’ directions far from the vertical category boundary (the second, third, sixth, and seventh directions on the right histograms of Figure 1d) were presented than on trials with ‘hard’ directions close to the vertical category boundary (the first, fourth, fifth, and eighth directions on the right histograms of Figure 1d). This pattern of task performance remained the same irrespective of motion direction variability (paired‐sample *t*‐tests on performance accuracy between ‘easy’ and ‘hard’ types of trials in all sessions, *p* < 10^−^^7^; see Table S1 for detailed statistics).

To examine the effect of motion direction variability on the behavioral performance on the motion categorization task, we plotted the proportion of trials in which mice turned the wheel to the “right” as a function of the mean motion direction in all three test sessions (Figure 1e–g). Separate cumulative normal functions were fit to each mouse's behavioral data for homogeneous (red graph in Figure 1e–g) and heterogeneous conditions (blue graphs in Figure 1e–g). We performed a one‐way ANOVA on the slope (1/standard deviation) of the normal curves to determine if the sensitivity to a mean motion direction was significantly influenced by the heterogeneity of the RDK (Figure 1h, *F_(16,80)_* = 11.02, *p* < 0.0001). Although mice had never been exposed to heterogeneous stimuli before the test session, the statistical test showed that the mice's sensitivity (1/σ) to a mean motion direction was similar between homogeneous and heterogeneous RDKs with motion direction ranges of 90° and 180° (90° session: Tukey's multiple comparison test: Hom − Het = 0.02, *p* = 0.997; 180° session: Tukey's multiple comparison test: Hom − Het = 0.08, *p* = 0.140). Overall behavioral performance across all eight global motion directions also remained broadly comparable between homogeneous and heterogeneous trials until the directional variability increased to 270° (90° session: 79.1% ± 0.06% vs. 79.2% ± 0.08%, *t_16_* = 0.13, *p* = 0.90, *d* = 0.02; 180° session: 80.1% ± 0.06% vs. 76.6% ± 0.1%, *t_16_* = 3.38, *p* = 0.004, *d* = 0.51; 270° session: 80.6% ± 0.05% vs. 68.3% ± 0.06%, *t_16_* = 9.88, *p* < 0.0001, *d* = 2.18). This finding of no significant difference in behavioral performance between homogeneous and heterogeneous conditions is surprising given that many different localized motion vectors of the individual dots span a very wide range of 90° or 180°. Only when the random dot motion vectors had an extremely large range of 270° from −135° to +135°, their sensitivity (1/σ) was significantly reduced compared to when the RDK was homogeneous (270° session: Tukey's multiple comparison test: Hom—Het = 0.20, *p* < 0.001). However, their overall performance across all eight mean motion directions of this 270°‐heterogeneous RDK was still significantly above chance. These results suggest that mice extract global mean motion directions from heterogeneous RDKs just as effectively as from homogeneous RDKs, and accurately assign them to the appropriate motion category.

Next, we further examined whether the degree of RDK heterogeneity differentially affected mice's sensitivity to the mean motion direction for easy and hard trials. By applying linear mixed‐effects model to *d′* values estimated for each heterogeneity level (see Experimental Section), we found that for both types of trials, *d′* values decreased with increasing RDK heterogeneity, more pronounced for easy (180°: homo *d′* − hetero *d′*  = 0.41, *t(176)* = 4.21, *p* = 4.08 × 10^−^^5^, *Cohen's d* = 1.44; 270°: homo *d′*  − hetero *d′*  = 0.86, *t(176)* = 8.93, *p* = 5.64 × 10^−^^1^ ^6^, *Cohen's d* = 3.06; Figure S1a) than hard trials (270°: homo *d′*  − hetero *d′*  = 0.38, *t(176)* = 3.96, *p *= 1.1 × 10^−^4, *Cohen's d* = 3.06; Figure S1b).

This result indicates that the heterogeneity level of RDKs has a higher impact on the sensitivity of mice to mean motion directions closer to the horizontal direction. However, even when the variance was as large as 90°, mice showed sensitivity comparable to that observed with homogeneous RDKs for both easy and hard trial types (Figure S1).

### 2.1 Single‐Cell Selectivity for Global Motion Direction in V1 and PPC

**FIGURE 2:**
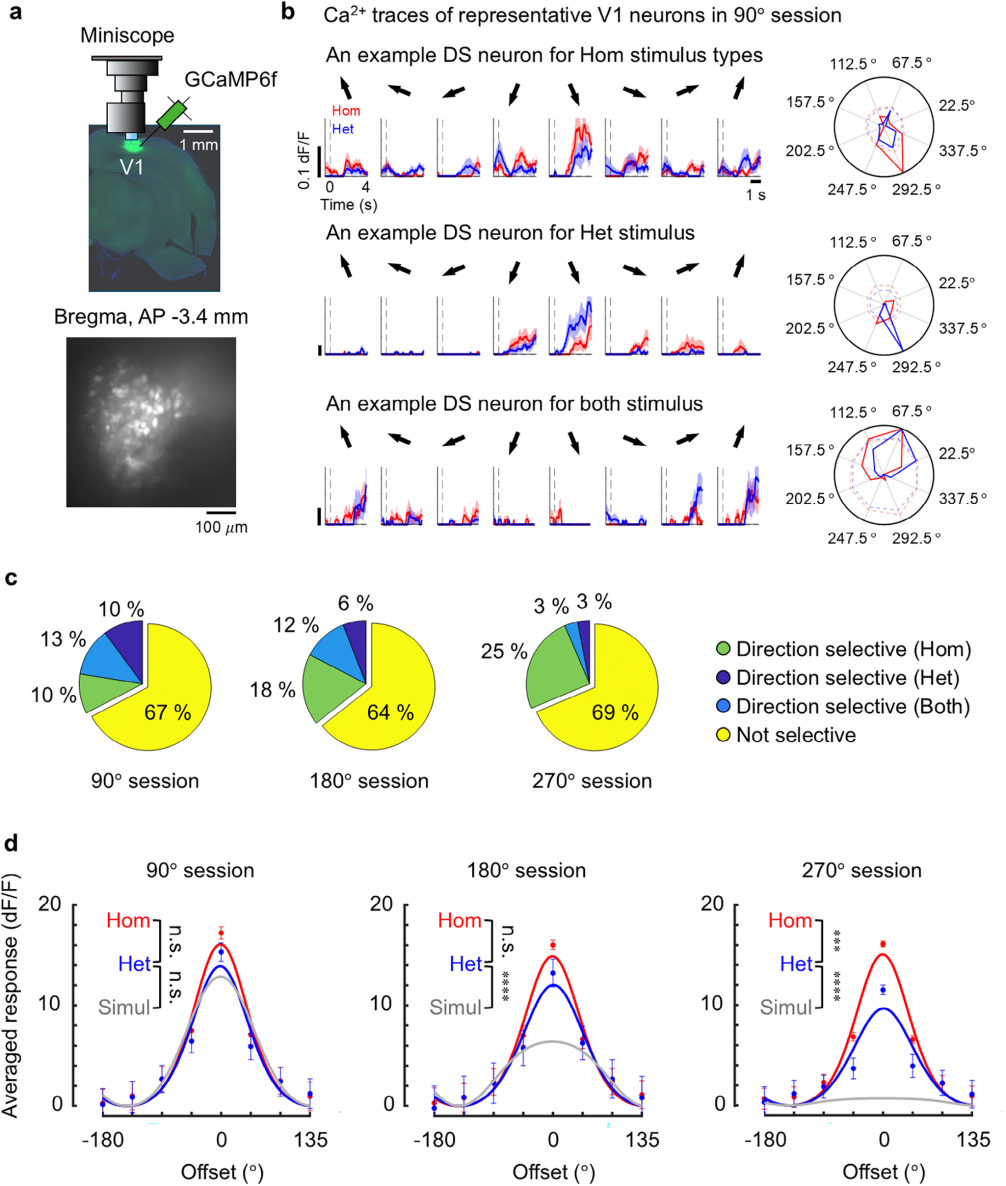
Global motion direction‐selective V1 neurons in response to homogeneous and heterogeneous RDKs during the motion categorization task. (a) (Top) One‐photon calcium imaging of L2/3 pyramidal V1 neurons expressing GCaMP6f. (Bottom) A frame‐averaged image of V1 neurons of a trained animal. (b) Representative stimulus‐evoked responses (*dF/F*) of V1 neurons showing various patterns of global motion direction tuning in 90° sessions—only homogeneous stimulus‐selective (first row), only heterogeneous stimulus‐selective (second row), and both homogeneous and heterogeneous stimulus‐selective (third row). The average signals of correct trials (up to 20) per global motion direction are displayed in red (Hom) and blue (Het). Shaded areas indicate ±SEM. Polar plots on the right of each response trace showed the AUC (area under the curve) of the Ca^2+^ response per global motion direction averaged over the 4 s of stimulus period. (c) Proportions of global motion direction‐selective V1 neurons in 90°, 180°, and 270° sessions. Global motion direction‐selective neurons were classified based on the direction selectivity index and the slope of the single neuronal tuning curve. Of all V1 neurons that passed the DSI threshold of 0.4, only neurons with slopes steeper than the top 5% of the slope distribution calculated from the trial‐shuffled dataset were considered as global motion direction‐selective neurons (5000 permutations). (d) Population‐averaged tuning curves of global motion direction‐selective V1 neurons in 90°, 180°, and 270° sessions. The red and blue curves indicate the tuning curve for homogeneous and heterogeneous RDKs, respectively. The gray curve is a simulated tuning curve for heterogeneous RDKs using the direction‐selective neurons identified in the homogeneous trials. The simulation was conducted 1000 times by resampling tuning curves of homogeneous RDKs, with the number of simulated neurons matched to the number of direction‐selective neurons observed in the heterogeneous condition for each of three test sessions. Data presented as mean ± SEM, 90° session (*n*_hom _= 295 *n*_het _= 295), 180° session (*n*_hom _= 418 *n*_het _= 245), 270° session (*n*_hom _= 386 *n*_het _= 88), *p*‐values were calculated using Welch's *t‐*test. ***p < 0.001, ****p < 0.0001.

To examine whether global mean motion directions were indeed encoded in the brain, we recorded calcium activity from V1 (Figure 2a) and PPC (Figure S2a) neurons using a UCLA Miniscope [32, 33] while 17 head‐fixed mice performed the motion categorization task. During the categorization task, we imaged a total of 1312, 1407, and 1367 neurons in layer 2/3 of V1 from 10 mice and a total of 997, 984, and 996 neurons in PPC from 7 mice in three test sessions with 90°, 180°, and 270° of directional variability, respectively. In order to investigate whether global mean motion directions were automatically represented in the brain without any confounds from decision‐making, motor, or reward signals accompanying task performance, we also recorded V1 and PPC activity while 14 head‐fixed mice passively viewed RDKs, imaging a total of 424, 674, and 523 neurons in layer 2/3 of V1 from 6 mice and a total of 1050, 1123, and 1006 neurons in PPC from 8 mice in three test sessions with 90°, 180°, and 270° of directional variability, respectively. These four groups of mice with different sample sizes were separated by task and recording area. Thus, during the motion categorization task or passive viewing, only within‐subject statistical comparisons between homogeneous and heterogeneous trials were performed in V1 and PPC, respectively.

To investigate whether neurons selective for global motion directions exist at all and, if so, how their selectivity changes as motion direction variability increases, we first performed conventional single‐cell‐level analyses by generating each neuron's direction tuning curves for homogeneous and heterogeneous trials, separately. For each of the eight global motion directions, we averaged the 4‐s poststimulus calcium signals and used the area under this average calcium signal (AUC) as the value of the single‐neuron tuning curve at each global motion direction. We focused our analyses on the data from correct trials to clarify the relationship between consistent behavior in response to a specific stimulus and the corresponding neural activity. We calculated the mean motion direction selectivity index (DSI; see Experimental Section). Of the neurons that exceeded the DSI threshold of 0.4 [34, 35, 36], only those in the top 5% of the slope distribution calculated from the trial‐shuffled dataset were classified as global motion direction‐selective neurons. Figure 2b shows representative V1 cells exhibiting three kinds of global motion direction‐selective responses (*dF/F*, averaged across correct trials) to homogeneous (red traces) and heterogeneous RDKs (blue traces) from one mouse in the 90° session. The first and second rows show global motion direction‐selective neurons in only one of the two types of trials. The third row shows one representative neuron displaying global motion direction selectivity in both types of trials. One‐third of all recorded V1 neurons displayed one of three kinds of global motion direction‐selective responses across both types of trials (Figure 2c). 13% of neurons (165 out of 1312) were direction‐selective in both types of trials during the 90° session (sky blue sector in the first column of Figure 2c). We also found that about 10% (129 and 136 out of 1312) of the neurons were direction‐selective in only one of the two RDK conditions, meaning that coherent motion direction‐selective neurons in homogeneous trials did not show selectivity for any mean motion direction in heterogeneous trials (green sector in the left column of Figure 2c), and vice versa (blue sector in the left column of Figure 2c). Similar results were observed in the 180° and 270° sessions, with the proportion of direction‐selective neurons tending to decrease progressively in the heterogeneous trials (blue sector in the middle and right columns of Figure 2c). Nevertheless, the proportion of neurons displaying direction selectivity in both trial types was significantly higher than the chance level of overlap between the direction‐selective neurons observed in each stimulus type. This was consistent across all three motion direction ranges (see Table S2 for detailed statistics). In contrast to V1 neurons, only a fraction of PPC neurons recorded from 7 mice exhibited global motion direction selectivity in all three test sessions (Figure S2b), but the proportion of direction‐selective PPC neurons in both types of trials was significantly higher than the chance level in all three ranges of motion direction just like V1 (Table S2).

In V1 and PPC, to visualize and compare the tuning sharpness of the direction‐selective neurons identified in the homogeneous and heterogeneous conditions, we summarized global motion direction‐selective responses at the single‐cell level by zero‐centering each neuronal tuning curve relative to its preferred global motion direction and averaging them across all direction‐selective neurons under homogeneous (red graphs) and heterogeneous (blue graphs) conditions, respectively (Figure 2d; Figure S2c). By comparing the peak amplitude of the population‐averaged tuning curves between homogeneous and heterogeneous conditions, we found that neuronal selectivity for global motion direction in V1 was remarkably similar between homogeneous and heterogeneous trials with motion direction ranges of 90° and 180° despite the fact that many different localized motion vectors of the individual dots span a very wide range of 90° and 180° (the left and middle columns of Figure 2d, red vs. blue). This is consistent with our surprising finding of no significant difference in behavioral performance between homogeneous and heterogeneous conditions in both 90° and 180° test sessions (Figure 1e,f). Only when the motion direction range of the heterogeneous RDK was 270°, the amplitude of the tuning curve was substantially reduced relative to that of the homogeneous RDK (the right column of Figure 2d, red vs. blue, Welch's *t*‐test, *t(183.3)* = 3.55, *p* = 0.0005). Similarly, in PPC, the population‐averaged tuning curves were not significantly different between homogeneous and heterogeneous conditions across all three test sessions (Figure S2c, red vs. blue; Welch's *t*‐test, 90° session: *t(188.5)* = 0.08, *p* = 0.93, 180° session: *t(168.9)* = 0.24, *p* = 0.81, 270° session: *t(95.35)* = 0.5167, *p* = 0.61). These observations suggest that V1 and PPC neurons encode the global mean motion direction rather than simply detect local motion directions of individual moving dots.

As noisy inputs such as a wide range of local motion directions exhibited by individual dots in heterogeneous RDKs are known to broaden tuning curves due to the reduced overall sensitivity or gain of a neuron (Figure S3; see Experimental Section for details), we investigated whether a tuning curve of a heterogeneous RDK could emerge from a linear summation of individual tuning curves corresponding to local motion directions within the range of the heterogeneous RDK. Thus, we simulated tuning curves for heterogeneous RDKs using the direction‐selective neurons identified in the homogeneous trials. In V1 and PPC, although the direction‐selective responses in the heterogeneous trials of the 90° session can be explained by linear summation of responses to individual moving dots, the amplitudes of the empirically observed tuning curves in the heterogeneous trials with 180° and 270° of direction variability were substantially higher than those of the simulated tuning curves (blue vs. gray; Figure 2d, Welch's *t*‐test, 180° session: *t(362.1)* = 4.36, *p* < 0.0001, 270° session: *t(104.1)* = 4.42, *p* < 0.0001; Figure S2c, Welch's *t*‐test, 180° session: *t(129.7)* = 2.54, *p* = 0.01, 270° session: *t(66.95)* = 2.37, *p* = 0.02). These findings indicate that both V1 and PPC neurons are much more sharply tuned to the global mean motion directions than would be expected from a simple linear summation of local motion vectors.

When applying the same single‐cell‐level analyses used in the motion categorization task to V1 and PPC calcium activity recorded during passive viewing, we obtained results similar to those in the above task conditions. We found that the distributions of global mean motion direction–selective V1 and PPC neurons (Figures S4a and S5a) were similar to those observed during motion categorization (Figure 2c; Figure S2b). Simulation analyses also showed the same pattern of results observed in the task conditions (Figure 2d; Figure S2c), confirming that a simple linear summation of local motion vectors was not sufficient to capture the sharp tuning curves of global mean motion direction‐selective V1 and PPC neurons observed during passive viewing (Figures S4b and S5b). These results suggest that mean motion direction‐selective neurons already exist in V1 in the absence of task demands.

### 2.2 Variability of Global Motion Direction‐Selective Neurons in V1 and PPC

**FIGURE 3:**
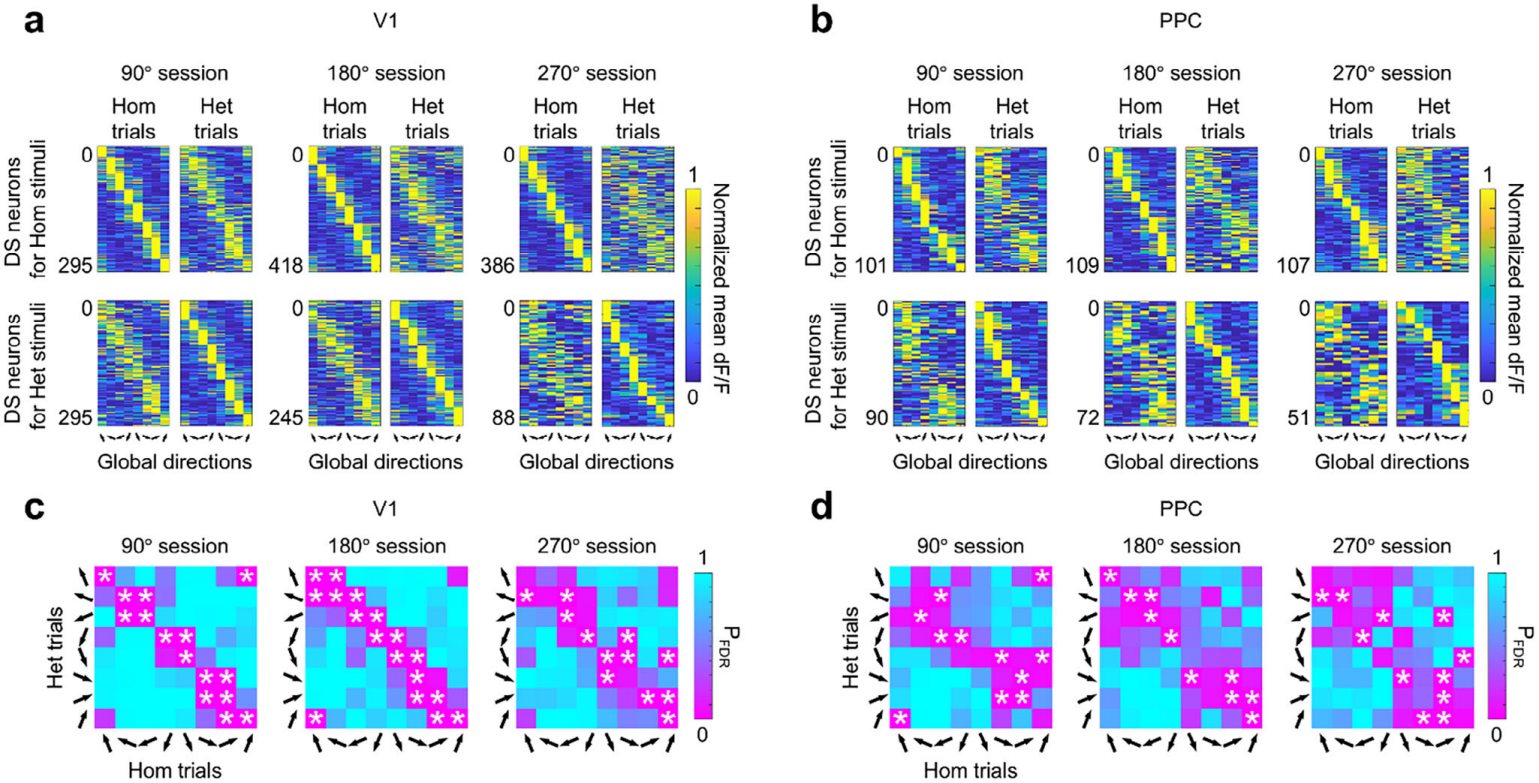
Variability of global motion direction‐selective V1 and PPC neurons during the motion categorization task. (a) The tuning curves of global motion direction‐selective V1 neurons in 90°, 180°, and 270° sessions (three columns of a 2 × 2 matrix of rectangular graphs). In each session, the top/bottom panels show how the tuning curves of the direction‐selective V1 neurons identified in the homogeneous (top‐left graph) / heterogeneous (bottom‐right graph) trials changed in response to heterogeneous (top‐right graph) / homogeneous (bottom‐left graph) RDKs. In each rectangular graph, the *x*‐axis indicates eight global motion directions of RDKs and the *y*‐axis indicates direction‐selective V1 neurons identified in homogeneous (top) or heterogeneous (bottom) trials, sorted by the peak positions of their tuning curves. Color values represent the tuning curve responses of the direction‐selective V1 neurons at eight global motion directions. (b) The tuning curves of global motion direction‐selective PPC neurons in 90°, 180°, and 270° sessions. The results are presented in the same format as (a). (c) The pairs of global motion directions of homogeneous and heterogeneous RDKs that elicited the maximal responses from all recorded V1 neurons in 90°, 180°, and 270° sessions. In each session, the color values at row *i* and column *j* represent the proportion of V1 neurons that responded maximally to the mean motion direction *i* of heterogeneous RDK and the coherent motion direction *j* of homogeneous RDK. (d) The pairs of global motion directions of homogeneous and heterogeneous RDKs that elicited the maximal responses from all recorded PPC neurons in 90°, 180°, and 270° sessions. The results are presented in the same format as (c). Total numbers of PPC neurons are 997, 984, and 996 for the 90°, 180°, and 270° sessions, respectively. Asterisk indicates the proportion of neurons that is significantly larger than the surrogate proportion of neurons obtained from randomly shuffling trials (5000 permutations, *p *< 0.05 based on FDR). Total numbers of V1 neurons are 1312, 1407, and 1367, and the total numbers of PPC neurons are 997, 984, and 996 for the 90°, 180°, and 270° sessions, respectively.

As we observed that the identities of V1 and PPC neurons that passed the direction‐selectivity criteria differed between homogeneous and heterogeneous trials (Figure 2c; Figure S2b), we investigated whether this was because neurons classified as selective for a specific global motion direction in one condition simply failed to pass the direction‐selectivity criteria in the other condition, or because they actually changed their preferred global motion direction (i.e., the direction of maximal response). We visualized how individual tuning curves of neurons selective for coherent motion directions of homogeneous RDKs changed as a function of the mean motion direction of heterogeneous RDKs (the top row of Figure 3a,b) and vice versa (the bottom row of Figure 3a,b). By quantifying the similarity of direction‐selective response patterns between homogeneous and heterogeneous conditions in each of the three sessions, we found that individual V1 neurons tended to prefer similar directions in both conditions. However, fewer V1 neurons preferred a similar global motion direction across the homogeneous and heterogeneous trials as the motion direction variability of the heterogeneous RDK stimulus increased (Figure 3a, 90° session: Pearson correlation coefficient *R* = 0.69, *p* < 0.05 between the top‐left and top‐right matrices and *R* = 0.65, *p* < 0.05 between the bottom‐left and bottom‐right matrices, 180° session: Pearson correlation coefficient *R* = 0.55, *p* < 0.05 between the top‐left and top‐right matrices and *R* = 0.64, *p* < 0.05 between the bottom‐left and bottom‐right matrices, 270° session: Pearson correlation coefficient *R* = 0.29, *p* < 0.05 between the top‐left and top‐right matrices and *R* = 0.32, *p* < 0.05 between the bottom‐left and bottom‐right matrices). We also observed that V1 neurons classified as tuned to a specific global motion direction of either homogeneous or heterogeneous RDK changed their global motion direction selectivity even when we randomly split trials in half within the homogeneous or heterogeneous RDK condition (Figure S6b,c).

Compared with V1 neurons, an even smaller proportion of PPC neurons exhibited mean motion direction selectivity (Figure S2b) and the direction‐selective response pattern of these PPC neurons was less correlated across homogeneous and heterogeneous conditions (Figure 3b, 90° session: Pearson correlation coefficient *R* = 0.48, *p* < 0.05 between the top‐left and top‐right matrices and *R* = 0.45, *p* < 0.05 between the bottom‐left and bottom‐right matrices, 180° session: Pearson correlation coefficient *R* = 0.44, *p* < 0.05 between the top‐left and top‐right matrices and *R* = 0.43, *p* < 0.05 between the bottom‐left and bottom‐right matrices, 270° session: Pearson correlation coefficient *R *= 0.40, *p *< 0.05 between the top‐left and top‐right matrices and *R* = 0.37, *p* < 0.05 between the bottom‐left and bottom‐right matrices). That is, rather than maintaining direction preferences, PPC neurons that preferred a specific direction under one condition tended to respond to all other directions within the same motion category (left or right) in the other condition. This variability of global motion direction‐selectivity in PPC neurons was also observed when trials were randomly split in half within the homogeneous or heterogeneous RDK condition (Figure S6d,e). These results suggest that PPC neurons may encode directional information differently from V1, potentially reflecting more categorical information.

Additionally, we comprehensively examined whether the preferred global motion direction remained the same in both homogeneous and heterogeneous trials using all recorded V1 neurons regardless of whether they passed the criteria for direction‐selective neurons. We tracked whether V1 neurons maximally responsive for a specific mean motion direction of the heterogeneous RDK showed a peak response to the same or different coherent motion direction of the homogeneous RDK. In this way, we constructed a confusion matrix whose elements were the number of V1 neurons exhibiting maximal responses at the corresponding pair of global motion directions of homogeneous and heterogeneous RDKs. A permutation‐based test with false discovery rate (FDR) correction across the resulting confusion matrix confirmed that a large proportion of V1 neurons remained sensitive to similar global mean motion directions across both types of trials as shown in significant diagonal elements of the confusion matrices in all three test sessions, although DSI analysis of single neurons was not consistently reliable across different trials (Figure 3c). This suggests that the majority of untuned cells in mouse V1 may still support sensitivity to the global mean motion direction at the neural population level.

Unlike V1, a large proportion of all recorded PPC neurons did not show peak responses at the same or neighboring global motion directions across both RDK conditions. In the confusion matrix, PPC neurons exhibiting peak responses were divided into two groups by the vertical category boundary without showing specificity for the pair of global motion directions from both types of trials (Figure 3d). These findings indicate that PPC neurons still retained the selectivity for the global motion direction in both types of trials, but their preferred global motion direction of homogeneous (or heterogeneous) RDK changed to one of the four global motion directions of heterogeneous (or homogeneous) RDK in the motion category to which they belong.

During passive viewing, we observed V1 and PPC neurons that were selective for global motion direction (Figure S7), indicating the existence of V1 and PPC neurons that automatically pool multiple local motion vectors into a global motion direction independently of task demands or decision‐related signals. However, their selectivity for global motion direction was variable across homogeneous and heterogeneous trials (Figure S7) as shown in the above task condition (Figure 3). These results suggest unreliable variability in global motion direction selectivity at the single‐cell level, regardless of task demand.

### 2.3 Neural Population‐Level Representations of Global Mean Motion Directions in V1 and PPC

**FIGURE 4:**
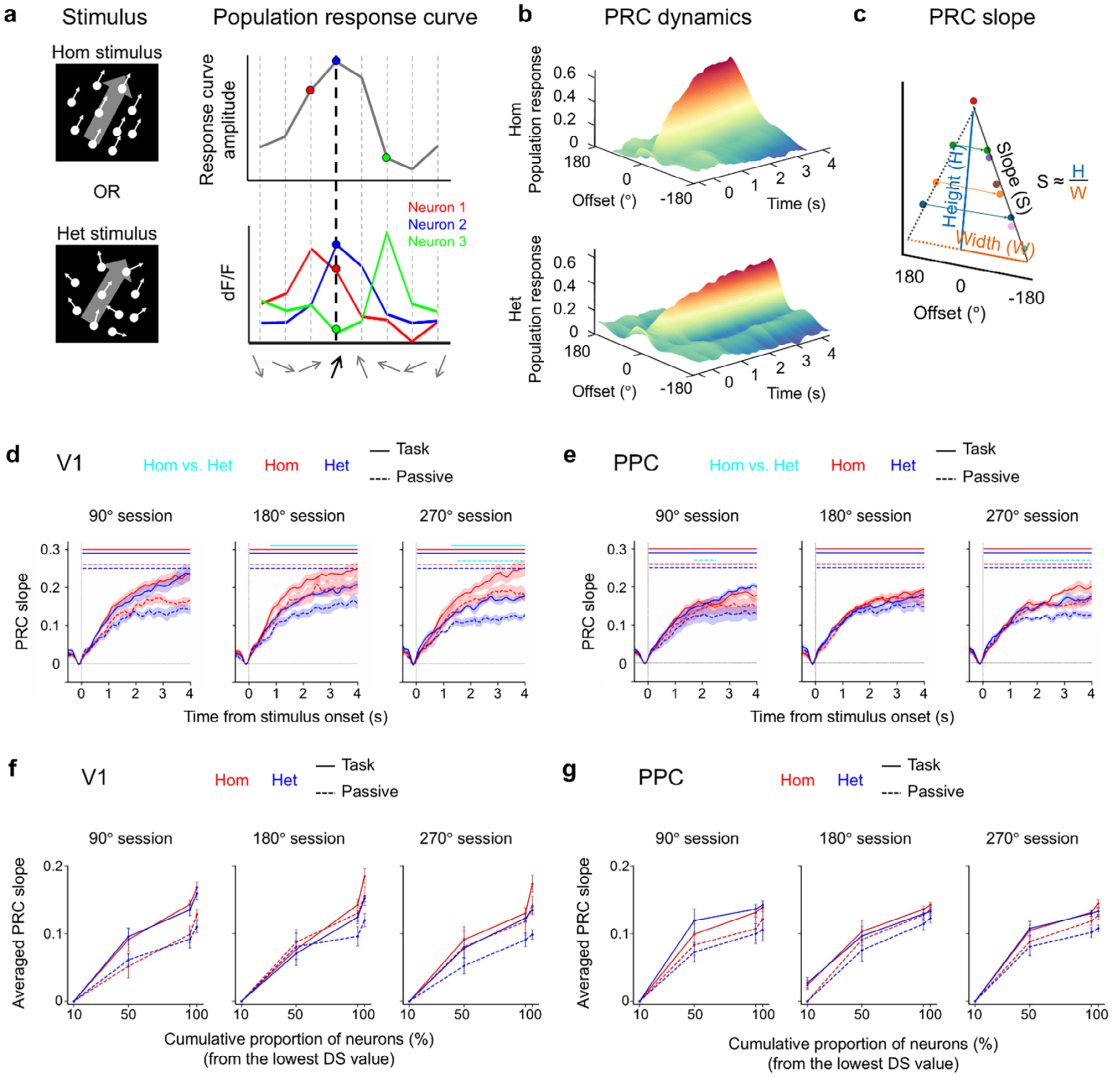
The global motion direction encoding in multivariate V1 and PPC activity. (a) Schematic population response curve (PRC) when the global motion direction of RDK is 67.5°. Single‐cell global motion direction tuning curves (red, green, and blue curves in bottom‐right) are obtained for homogeneous (top‐left) and heterogeneous RDKs (bottom‐left) separately. When neuronal tuning curves center at three different locations in motion direction space, the gray curve shows how these neurons respond to the RDK presented at 67.5° of global motion direction (red, blue, and green dots in top‐right and bottom‐right). In the schematics, only one neuron per the preferred global motion direction is shown. (b) Population response curve dynamics. The population response curves constructed at every time point show that the population responses are sharply modulated after the stimulus onset. Top, PRC for homogeneous RDK. Bottom, PRC for heterogeneous RDK. (c) Population response curve slope. PRCs tuned to global motion directions are summarized by calculating the slopes of PRCs. Specifically, we first reversed the sign of motion directions. We then calculate the slopes by fitting the linear regression with an intercept. (d,e) PRCs in 90°, 180°, and 270° sessions for V1 (d) and PPC (e). The encoding accuracies of the global motion directions are sharply increased after the stimulus onset. Red and blue colors indicate homogeneous and heterogeneous trials, respectively. Shaded areas indicate ±SEM. Solid and dashed lines above the figures indicate the time period when the PRC slope is significantly greater than 0 (*p *< 0.05, based on cluster extent) during the motion categorization task and passive viewing, respectively (Table S3). Cyan solid and dashed lines above the figures indicate the time period when the PRC slope between homogeneous and heterogeneous trials is significantly different (*p *< 0.05, based on cluster extent) during the motion categorization task and passive viewing, respectively (Table S3). (f,g) PRC slopes averaged over the 4 s post‐stimulus period as a function of the number of neurons in 90°, 180°, and 270° sessions for V1 (f) and PPC (g). Using the bottom 10%, 50%, 95%, and 100% of all recorded neurons that are rank‐ordered by their single‐neuron tuning curve slopes, tested against slopes of the trial‐shuffled dataset, the average PRC slopes are calculated. Red and blue colors indicate homogeneous and heterogeneous trials, respectively. Error bars indicate ±SEM. Data presented as the mean ± SEM, V1 task *N* = 10, V1 passive *N* = 6, PPC task *N* = 7, PPC passive *N* = 8, statistical analysis by cluster‐based permutation test (two‐tailed; cluster‐forming threshold *p* < 0.05, uncorrected; cluster‐mass statistic; 5000 permutations; FWER *α* = 0.05). Significant clusters are indicated by bars above the trace.

We found considerable trial‐to‐trial variability in global motion direction selectivity at the single‐cell level during both the categorization task and passive viewing. This observation suggests that not only classified mean motion‐selective neurons, but also the majority of neurons not classified as direction‐tuned, may contribute to global mean motion processing at the population level (Figure 3; Figure S7). Based on this, we examined whether multivariate V1 and PPC activity patterns robustly encoded global mean motion direction. We constructed a population response curve (PRC) [37, 38, 39] from multivariate activities recorded during both the motion categorization task and passive viewing (Figure 4a; Figure S8a; see Experimental Section for details). The PRC represents the collective activity of a group of neurons in response to a given global motion direction of homogeneous (top‐left of Figure 4a) or heterogeneous RDK (bottom‐left of Figure 4a). For a given RDK (the black arrow in the bottom‐right of Figure 4a), the responses of all neurons that prefer the presented global motion direction are averaged across neurons and trials (the blue curve in the bottom‐right of Figure 4a), resulting in the value of the PRC at the presented global motion direction (the blue point in the top‐right of Figure 4a). In the same way, the values of the PRC at other global motion directions are obtained for a given RDK: the responses of neurons that prefer the global motion direction other than the presented one are averaged across those neurons and trials (the red and the green curves in bottom‐right of Figure 4a), resulting in the value of the PRC at other global motion directions (the red and green points in top‐right of Figure 4a). All recorded neurons were included in constructing the PRC, regardless of whether they met the selectivity criteria. Thus, when the response of each neuron to a single fixed global motion direction is plotted, the resulting curve across the population is the PRC. This procedure was repeated for all time points in every homogeneous and heterogeneous RDK stimulus epoch, respectively. This is distinct from a population‐averaged tuning curve, which shows how a single neuron responds to varying global motion directions (Figure 2d; Figure S8b). Finally, every PRC was zero‐centered relative to the presented global motion direction and averaged across trials for the homogeneous and heterogeneous conditions separately (Figure 4b). We summarized PRC dynamics by calculating the linear slope of the zero‐centered PRC at each time point in the epoch (Figure 4c; see Experimental Section for details). The higher PRC slope corresponded to greater global motion direction selectivity, while the zero PRC slope corresponded to no global motion direction selectivity. Multiple comparisons across time points were corrected using non‐parametric cluster‐based permutation testing with a significance level of 0.05 [40].

During both the motion categorization task and passive viewing, global mean motion direction was accurately represented in multivariate V1 and PPC activities for almost the entire period following homogeneous and all three heterogeneous RDKs (Figure 4d,e; See Table S3 for detailed statistics). In V1, PRC slopes were not significantly different between homogeneous and heterogeneous RDK with a range of 90° regardless of task demand (the first column of Figure 4d). However, as the motion direction variance of RDK increased, significant differences in PRC slopes between the homogeneous and heterogeneous conditions emerged (solid and dashed cyan lines in the second and third columns of Figure 4d). We also observed that neural representations of mean motion directions in V1 were less accurate when mice passively viewed RDKs compared with when mice were actively engaged in the motion categorization task. In PPC, the accuracy of mean motion direction encoding showed no significant differences between homogeneous and heterogeneous conditions at any level of motion direction variance during the motion categorization task (Figure 4e). Even when trials were randomly split in half, the population‐level PRC showed robust global motion direction encoding in V1 and PPC (Figure S9), even though individual neurons exhibited variability in global motion direction selectivity (Figure 3a,b; Figure S7a,b). These results suggest that neural responses of the majority of unclassified mean motion direction‐selective cells in mouse V1 and PPC are aggregated to robustly support a sensitivity to the global mean motion direction at the neural population level.

Next, to assess the extent to which neurons not classified as global motion direction‐selective contribute to encoding global motion direction, we first ranked all recorded neurons by their tuning curve slopes tested against a permuted slope distribution. We then calculated the PRC slopes using a subset of neurons in the bottom 10%, 50%, 95%, and 100% of the permuted slope distribution (Figures S10 and S11). The PRC slopes averaged over the 4 s post‐stimulus period revealed significant PRC slopes even when only using the bottom 50% of neurons (Figure 4f,g), indicating that neurons that did not pass the criteria to be classified as direction‐selective could still substantially contribute to the precise enough encoding of global motion direction information in both V1 and PPC.

Furthermore, we examined whether the robust neural population‐level representation of the global mean motion direction in V1 and PPC was predictive of behavioral performance. We found that the PRC slope was positively correlated with behavioral performance in V1, but not in PPC (V1: *r* = 0.51, *p* = 0.00074, *CI* = [0.24, 0.71]; PPC: *r* = 0.23, *p* = 0.23, *CI* = [−0.09, 0.51]; Figure S12a). When we assessed the correlation between the difference in average PRC slopes between homogeneous and heterogeneous trials and the corresponding difference in the slope of psychometric functions in Figure 1, a similar result was observed (V1: *r *= 0.37, *p* = 0.04, *CI* = [0.07, 0.61]; PPC: *r* = 0.31, *p* = 0.18, *CI* = [0, 0.57]; Figure S12b). These findings indicate an association between category‐based decision‐making performance of the mice and neural population‐level global motion direction encoding in V1.

### 2.4 Mean Motion Direction Representation Biased Toward the Category Center in V1 due to Category Learning

**FIGURE 5:**
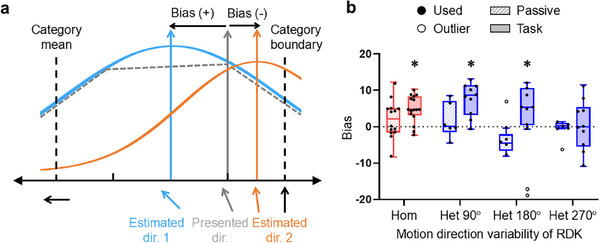
Categorical bias in global motion direction encoded in multivariate V1 activity. (a) Illustration of quantifying categorical biases in PRCs. For the presented global motion direction of RDK, the PRC averaged over the 4 s post‐stimulus period was fit with a wrapped normal function. Neural bias was measured by subtracting the estimated (sky blue or orange) from the presented (gray) global motion direction. When this difference is positive (or negative), category bias manifests as a shift toward the category center (or boundary). (b) Task‐dependent category bias in V1 as a function of motion direction variability of RDK. See Table S4 for detailed statistics. White circled outliers (>1.5 × interquartile range) were excluded to improve the statistical reliability. Data presented as the mean ± SEM, task *N* = 10, passive *N* = 6, statistical analysis by paired *t*‐test with FDR correction. *p < 0.05, **p < 0.01, ***p < 0.001, ****p < 0.0001.

According to previous studies [41, 42], category learning distorts perceptual sensitivity for category‐defining features such that differences between physically similar yet categorically distinct exemplars are enhanced, whereas differences between stimuli from the same category are reduced. Thus, we examined whether perceptual distortions following category learning are reflected in neural representations of mean motion directions in V1 activity patterns. In order to assess the top–down impact of the task demand on neural representations of mean motion directions, we averaged PRCs over the period from 0 to 4 s after the stimulus onset and fit the resulting PRC with a wrapped‐normal function (see Experimental Section for details). We then calculated the difference between the to‐be‐categorized mean motion direction of the RDK and the estimated center obtained from the PRC fit in both passive viewing data and motion categorization task data (Figure 5). This difference served as a measure of categorical bias toward the category center in the neural representation of global mean motion direction. Positive values indicate a bias toward the horizontal category center, whereas negative values indicate a bias toward the vertical category boundary (Figure 5a). We found that V1 exhibited no categorical bias when mice passively viewed homogeneous as well as heterogeneous RDKs (striped bar graphs in Figure 5b; Table S4 for detailed statistics). When mice performed the motion categorization task, significant categorical biases emerged in both homogeneous and heterogeneous conditions (filled bar graphs in Figure 5b; Table S4 for detailed statistics). These findings suggest that task‐induced top–down influences may modulate the neural representation of mean motion direction even in V1, an early stage of cortical visual processing.

### 2.5 Neural Encodings of Increasingly Abstract Features From Summary Statistics to Categories Across the Cortical Hierarchy

**FIGURE 6:**
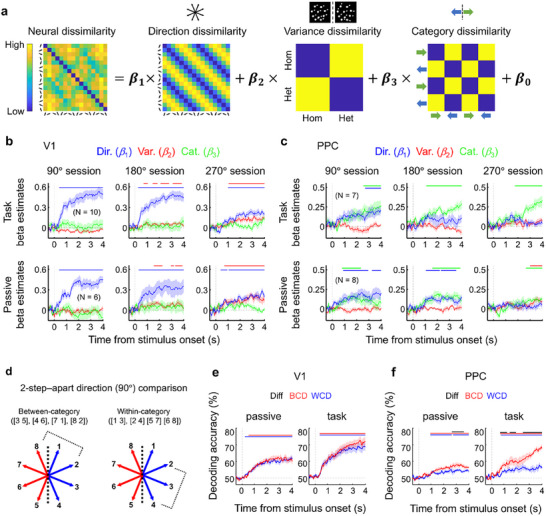
Neural encoding of mean, variance, and category similarity in V1 and PPC. (a) For the RSA, at each time point, the neural RDM was constructed by calculating the pairwise decoding accuracy for all pairs of stimuli. This neural RDM was then modeled as a linear combination of mean, variance, and category RDM. Mean RDM indicates the similarity between inputs. Variance RDM indicates the homogeneity of each input. Category RDM indicates the category similarity between inputs. (b) Time course of three regression coefficients in V1. Model RDMs labeling the symbolic number within each of the three means, variance, and category were regressed against the time course of neural activity within each trial. Each of the three coefficient time courses resulting from regression against each of the mean, variance, and category RDM is shown for three tasks (top) and passive (bottom) sessions. The cluster‐corrected significance at each time point is indicated along the top of each figure. Shaded areas indicate ±SEM. (c) Time course of three regression coefficients in PPC. The results are presented in the same format as (b). (d) Example of BCD and WCD calculations. From the 8‐direction set, a pair of mean directions that were 90° apart was chosen. Between‐category discrimination (BCD) was performed across the four pairs of directions that were 90° apart and crossed the category boundary (e.g., 1 vs. 7, 2 vs. 8, 3 vs. 5, and 4 vs. 6). Within‐category discrimination (WCD) was performed across the four pairs of directions that were 90° apart and lay within the same category (e.g., 1 vs. 3, 2 vs. 4, 5 vs. 7 and 6 vs. 8). (e,f) BCD versus WCD in V1 (e) and PPC (f). For each direction pair, a time‐resolved binary SVM classifier was used to discriminate between the two directions, and decoding at each time point was performed using repeated stratified fivefold cross‐validation with 100 repetitions (chance = 50%). Curves show WCD accuracy (blue) and BCD accuracy (red). Black lines over the graphs indicate time points where BCD accuracy was significantly higher than WCD accuracy (cluster‐ corrected *p* < 0.05), indicating the dominance of category information. Time is aligned to stimulus onset (0 s); dotted lines mark stimulus onset. Data presented as the mean ± SEM, V1 task *N* = 10, V1 passive *N* = 6, PPC task *N* = 7, PPC passive *N* = 8, statistical analysis by cluster‐based permutation test (two‐tailed; cluster‐forming threshold *p* < 0.05, uncorrected; cluster‐mass statistic; 5000 permutations; FWER *α* = 0.05). Significant clusters are indicated by bars above the trace.

Given that environmental volatility and categorical bias are reflected in neural representations of mean motion direction, we directly probed how mean motion direction, motion direction variance, and motion category each contributed to V1 and PPC activity patterns by modeling the neural representational dissimilarity matrix (RDM) [43, 44] as a linear combination of mean, variance and category dissimilarity (Figure 6a; see Experimental Section). This regression‐based RSA allowed us to independently track the neural coding dynamics of increasingly abstract features from summary statistics to categories along the cortical hierarchy.

Figure 6b showed that almost the entire time points from stimulus onset were dominated by the mean motion direction information in V1 activity patterns (blue lines in the top and bottom rows of Figure 6b; Table S5 for detailed statistics). Despite the categorical bias in V1 (Figure 5b), the absence of category dissimilarity suggests that distinct patterns of V1 activity mainly encode eight mean motion directions. The presence of the variance information in V1 activity patterns (red lines in the top and bottom panels of Figure 6b; Table S5 for detailed statistics) indicates that multivariate activity in mouse V1 is well suited to process summary statistics such as mean and variance.

In contrast, motion category dissimilarity contributed robustly to neural dissimilarity in PPC activity patterns when mice performed the motion categorization task (green lines in the top row of Figure 6c; Table S5 for detailed statistics). Category selectivity appeared around 1 s after stimulus onset and kept increasing until the end of the trial across all three task sessions. The global motion direction information appeared only in the 90° session (blue lines in the top row of Figure 6c; Table S5 for detailed statistics). These results are consistent with previous studies reporting that PPC is involved in processing stimulus categorization during perceptual decision making [28, 29, 30, 31, 45, 46, 47]. During passive viewing, however, the category information was intermixed with global motion direction information in both 90° and 180° sessions (green and blue lines in the bottom row of Figure 6c; Table S5 for detailed statistics). The gradual increase of the category information toward the end of the trial was not observed in any of the three passive viewing sessions unlike the motion categorization sessions.

As regression coefficients for variance and category information were weak, despite being statistically significant in Figure 6, we corroborated the results of RSA by applying time‐generalized multivariate pattern analysis [48] (i.e., temporal generalization (TG) method) to calcium signals in V1 and PPC in order to separately track the emergence of mean, variance, and category information over time (see Experimental Section; Figures S13–S15 for details). During motion categorization and passive viewing, we found that sustained activity patterns robustly encoded mean motion direction, motion variance, and motion category in V1 and PPC (see rectangle‐shaped significant time clusters of TG maps in Figures S13–S15 and different color lines of significant time points over Figures S13–S15). There were three differences compared with the RSA results—(1) PPC activity pattern discriminated between homogeneous and 270°‐heterogeneous RDK during motion categorization (Figure S14b compared with the top row, third column of Figure 6c). (2) PPC activity pattern robustly encoded mean motion direction in the 180° and 270° sessions during motion categorization and in the 270° session during passive viewing (Figure S13b compared with Figure 6c), which was consistent with PRC slope dynamics in Figure 4e. (3) V1 activity pattern robustly discriminated between the two motion categories of homogeneous, 90°‐, 180°‐, and 270°‐heterogeneous RDKs during both motion categorization and passive viewing (Figure S15a compared with Figure 6b).

As category decoding in V1 and mean motion direction decoding in PPC were most likely due to collinearity problem between the two RSA predictors, mean motion direction and motion category, we investigated which of the two was dominantly encoded in each brain area by comparing the decoding accuracy of within‐category mean direction and that of between‐category mean direction [28] (Figure 6d–f). Specifically, from the 8‐direction stimulus set, we chose a pair of mean directions that were 90° apart and discriminated each pair of mean directions using a binary support vector machine (SVM). The SVM decoder was trained and tested on homogeneous trials with repeated stratified five‐fold cross‐validation (see Experimental Section; Figure 6d). The SVM decoder trained on homogeneous trials was applied to heterogeneous trials. Within‐category discrimination (WCD) was performed across the four pairs of mean directions that were 90° apart and stayed within the same category (1 vs. 3, 2 vs.7, 5 vs. 7 and 6 vs. 8). Between‐category discrimination (BCD) was performed across the four pairs of mean directions that were 90° apart and crossed the category boundary (1 vs. 7, 2 vs. 8, 3 vs. 5 and 4 vs. 6). Both WCD and BCD accuracies were calculated separately for homogeneous, 90°‐, 180°‐, and 270°‐heterogeneous trials. We then averaged the WCD accuracies and BCD accuracies across four levels of motion variance and used them to evaluate their difference between WCD and BCD in V1 and PPC. This comparison of WCD and BCD revealed a significant difference in PPC, but not in V1 during both passive viewing and motion categorization (Black lines over Figure 6e,f; Table S6 for detailed statistics), indicating that mean motion direction and motion category were dominantly encoded in V1 and PPC, respectively. Taken together, these results resolve the collinearity between the RSA predictors—mean motion direction and motion category—and confirm the RSA findings reported in Figure 6b,c.

## 3 Discussion

### 3.1 Concurrent Encodings of Ensemble Mean and Ensemble Variance in Mouse V1

Our study reveals that the mouse visual system encodes summary statistics of motion—specifically, the ensemble's mean direction and variance—at the earliest cortical stage, V1. By adopting RDKs in which dot directions were uniformly distributed around a controllable mean, we decoupled global summary statistics from the strong local cues present in conventional RDKs that consist of a subset of dots coherently moving in a specific direction and the rest of the dots moving in random directions. Consequently, neuronal selectivity observed in this context reflects integration over multiple local motion vectors, rather than simple detection of a prominent sub‐population of coherently moving dots. Unlike traditional RDKs that conflate mean and coherence, our uniformly distributed motion stimuli orthogonalize these parameters: increasing the dispersion widens the variance without strengthening any specific local cues. This stimulus, therefore, forces neuronal populations to pool across directions to recover the mean, while simultaneously signaling the uncertainty encoded by variance. The design thus offers a clean physiological assay for studying how and where the brain extracts first‐order and second‐order summary statistics of the visual scene.

The blunter tuning curves under high variance RDKs likely reflect increased sensory noise, but the observed tuning in V1 remained considerably sharper than expected from a simple linear summation model, suggesting that V1 does more than passively relay input (Figure 2d). Despite the absence of a dominant motion cue, about 33% of V1 neurons displayed global mean motion direction selectivity (Figure 2c), comparable to prior studies using plaid stimuli [49]. This early emergence of first‐order and second‐order summary statistical encoding in V1 is supported by the anatomy of the mouse visual system. The entire retina of the mouse is similar to the rod‐dominated peripheral retina of primates, specialized for low‐acuity, peripheral vision in dim light [50]. Without a fovea and with a retina rich in direction‐selective ganglion cells (DSGCs) covering ∼6° of visual space, mice are highly efficient at sampling and detecting local motion of the visual scene [51]. With V1 receptive fields spanning ∼20° and integrating over broad spatial extents, neurons in V1 are anatomically positioned to pool input from multiple DSGCs [52], naturally computing motion summaries of local motion over a substantial area such as the expected direction and the uncertainty of motion distribution. This is particularly relevant for encoding coarse‐grained information such as optic flow or motion reliability, where fine detail is costly to resolve and large‐scale motion patterns provide important behavioral cues. Our PRC analysis revealed reliable, bell‐shaped tuning curves across all sessions and behavioral contexts. Under both passive viewing and active categorization, multivariate activity patterns discriminated global mean motion directions with high fidelity, and the slope of the PRC declined systematically with increasing variance (blue graphs in three columns of Figure 4d), mirroring known psychophysical observations in ensemble perception [7, 53]. The model‐based RSA further confirmed robust encoding of mean motion direction (Figure 6b), while variance information appeared selectively when stimulus variance was high during both passive viewing and motion categorization (the second and third columns of Figure 6b; Figure S14a,c). The variance information in V1 might be due to task difficulty or attentional demand as more heterogeneous RDKs could impose greater cognitive load on integrating many local motion vectors into the mean motion direction than homogeneous ones. The fact that variance decoding accuracy during passive viewing was comparable to that during the categorization task (Figure S14a,c) suggests that the same cognitive load is placed on automatic integration of many local motion vectors into global mean motion direction regardless of the task demand. Another possibility is that the large receptive field of mouse V1 (∼20°) may allow V1 to directly and reliably represent the degree of heterogeneity of the scene itself. In either case, V1 is not a very sensitive integrator or detector, given that it cannot distinguish a homogeneous RDK from a 90°‐heterogeneous RDK. Despite the task relevance of categories, V1 showed no categorical encoding (Figure 6e), suggesting that its representations remain tied to summary statistical features even in behaviorally demanding contexts. These findings support a distributed encoding model in V1, where the population‐level representations remain robust and precise (Figure 4d; Figure 6b; Figure S13), even if single‐neuron selectivity appears weak (Figure 3a; Figures S6b,c and S7a).

Previous human fMRI studies have shown that mean feature information of the ensemble is represented in various brain regions along the visual hierarchy depending on the type of feature—mean orientation representation in occipital, parietal, and frontal cortex, but not in V1 [54] and mean facial expression representation in parietal and frontal cortex [55]. One monkey single‐cell recording study has also found evidence that MT encodes mean motion direction by integrating population responses to multiple directions of motion in MT [24]. These previous human and non‐human primate studies strongly indicate that the relatively small receptive fields of human and monkey V1 are optimized for fine‐grained, detailed visual processing, making V1 less suitable for representing summary statistics. This is probably why areas with larger receptive fields in higher cortical areas outside the early V1 area are more capable of such integration based on the pooled signals from multiple V1 receptive fields [56]. Thus, the observed coexisting mean and variance representations in mouse V1 may arise from its unusually large receptive fields, making mouse V1 suitable for coarse‐grained visual information such as mean and variance. Together, these studies suggest that pooling operations that integrate sufficient amounts of information can naturally code summary statistics at various hierarchical levels of the cortex [25]. Interestingly, prior research suggests that a similar principle of statistical pooling and summary encoding may underpin texture perception, which also exploits statistical regularity and element density rather than detailed element‐wise features in the visual scene [23, 57].

### 3.2 Categorical Biases in Ensemble Mean Representations in V1

While V1 did not explicitly encode category structure, we observed a systematic bias of mean motion direction representations toward the category center only during the motion categorization task (Figure 5b). This task‐dependent categorical warping aligns with a previous human neuroimaging study showing categorical distortion in early visual areas [41]. The lack of bias during passive viewing implies that the observed biases are not due to intrinsic oblique effects [58] but emerge as a result of task‐dependent category learning. This suggests that mouse V1 can exhibit flexible encoding schemes where summary statistical information abstracted from concrete sensory signals is modulated by top–down information about a more abstract category structure, while preserving higher‐order scene statistics of the underlying stimulus.

### 3.3 Neural Encodings of Ensemble Mean and Category in PPC

PPC also encoded global motion direction at both the single‐neuron (Figure S2) and population levels (Figure 4e,g), albeit with differences from V1. First, the fraction of direction‐selective PPC neurons was smaller (Figure S2b), and their preferred direction varied more across stimulus types (Figure 3b,d). Interestingly, these shifts remained within the same motion category, suggesting that PPC re‐encodes mean direction in a format aligned with categorical decision‐making [30]. Second, the PRC constructed from the bottom 50% of untuned neurons remained informative (Figure 4g; Figure S11), reinforcing the population coding architecture as in V1 (Figure 4f; Figure S10) [19, 20, 21, 22, 23]. But PPC's encoding accuracy for global motion direction was comparable for homogeneous and heterogeneous stimuli, suggesting a decision‐stage readout that cares about what the ensemble summary is, not how it arises. Third, unlike the PRC analysis, RSA showed that mean information appeared intermittently during passive viewing, but not during task sessions with large stimulus variance. In contrast, the sustained increase of category information toward the end of the trial was observed during active tasks but not during passive viewing. This late‐ramping category signal is highly likely related to preparatory activity or motor planning for decision making (Figure 6c) as PPC is known to be a hub for transforming sensory signals into motor signals, decision‐related variables encoding action choice in perceptual and memory‐guided tasks [59, 60]. However, our additional category‐specific decoding analysis showed that stable category information already appeared within 0–1 s after stimulus onset (Figure 6f; Figure S15b), which is temporally distinct from canonical motor‐preparatory signals that typically appear about 0.5–1 s before the behavioral response [61]. Thus, although the late ramping category signal could include signals reflecting motor/choice preparation during the task, the initial category signal is likely to represent pure categorical information. These observations suggest that PPC representations of global motion direction may depend on upstream input from V1 and be replaced by more abstract encodings especially during task performance [30].

The discrepancy between the PRC and RSA results may arise because the former isolates mean motion direction encoding within each motion variance, while the latter considers a representational space spanning direction, variance, and category. Within this broader space, PPC activity was dominated by category‐related patterns (Figure 6f; Figure S15b), reflecting its role in integrating noisy sensory input and transforming it into an abstract categorical code that facilitates decision‐making [28, 29, 30, 31, 45, 46, 47]. During passive viewing, category information likely reflects long‐term prior knowledge shaped by the prevalence of horizontal optic flow directions in natural scenes [62, 63]. Under task demands, however, long‐term priors [47, 64] of optic flow and short‐term evidence [59, 65] of category mean align, leading to the gradual increase in category information toward the end of the trial [66]. Thus, in both task and passive conditions, PPC appears to integrate bottom–up likelihoods for mean and variance from V1 with the existing representations of previous category knowledge and task demands to yield a posterior estimate that efficiently guides behavior [15], which may effectively bias the individual mean motion directions toward the category mean [30]. This probably explains why variance information was weak or absent in PPC during the active task—once the first‐ and second‐order summary statistics such as mean and variance are already processed in V1, it appears that variance‐dependent mean motion direction information relayed to PPC is enough to efficiently guide categorical choice behavior. Variance information observed in V1 during both the 180° and 270° sessions (Figure S14a,c) appeared to be transmitted to the PPC only during the 270° session (Figure S14b,d), leading to a sharp decline in category discrimination performance. These observations that behavioral performance was impaired and variance information in PPC was reliably decoded only in the 270° session raise the possibility that PPC activity patterns may reflect task difficulty or attentional demand rather than variance per se. However, we also cannot rule out the possibility that the variance decoding results observed in V1 and PPC may be confounded by session effects (as each homogeneity level was presented in a different session) and by differences in the neuronal populations recorded across sessions (with only ∼25% overlap).

### 3.4 Population Coding Enables Abstraction

One striking finding is that the encoding accuracy of global motion direction in both V1 and PPC relies not only on sharply tuned neurons but also on the broader population, including those with weak or ambiguous tuning. Even neurons with little global motion direction selectivity at the single‐cell level contributed to a precise population code; including only half of the nominally untuned neurons in the PRC analysis barely degraded the ensemble summary statistical representation (Figure 4f,g; Figures S10 and S11). This supports recent views that untuned or weakly tuned neurons carry significant stimulus information when read in concert with their neighbors [67, 68, 69]. Such a population coding scheme not only allows for efficient readout of summary statistics by improving the signal‐to‐noise ratio but also simplifies downstream transformations into categorical decisions. As a result, neurons that appear marginally tuned in isolation are essential contributors to the brain's capacity to extract reliable and behaviorally relevant patterns from noisy input. Moreover, population coding supports computational flexibility. Different behavioral contexts may emphasize different readouts (e.g., mean, variance, category), and a population‐level code allows selective access to whichever variable is most relevant. This capability may be especially critical in natural environments where sensory information is noisy and behavioral priorities change dynamically.

### 3.5 Limitations and Future Directions

While our multilevel analyses provide convergent evidence for hierarchical encoding and categorical abstraction across cortical areas, the present conclusions are derived primarily from multivariate pattern analyses. Thus, the current findings should be interpreted as correlational rather than causal. Future studies will benefit from incorporating causal manipulations—such as optogenetic or chemogenetic inactivation, local perturbation, or projection‐specific disconnection/isolation—to establish the current findings of hierarchical summary statistics encoding across cortical areas. Furthermore, combining such perturbation approaches with simultaneous calcium imaging could reveal how hierarchical representations reorganize in real time during task performance, illuminating the circuit‐level computations underlying summary‐statistic encoding in the visual cortex.

### 3.6 Summary and Implications

Our study advances three key points. First, the mouse visual cortex encodes both ensemble mean and variance at the earliest cortical stage, leveraging a rod‐dominated visual system to efficiently pool motion signals. Second, these summary statistical representations in V1 are relayed to PPC, where they are restructured into more abstract categorical representations for decision‐making [28, 30, 45, 46, 47]. Third, both V1 and PPC exhibit the power of population codes; even untuned neurons contribute significantly to precise summary statistical representations when considered collectively.

The coexistence of ensemble mean information and categorical encoding in PPC suggests that intermediate representations are maintained alongside more abstract ones. The brief presence of mean direction coding in PPC may reflect its dependence on V1 input and its role in binding summary statistics to categorical labels, consistent with the Bayesian inference framework that current sensory evidence is fused with an internal model of the world [62, 63]. The observed categorical biases in V1 and the decision‐aligned encoding in PPC are both compatible with this view of sensory processing. Thus, the hierarchical transition from summary statistical features in V1 to abstract categorical signals in PPC exemplifies a multi‐stage computation in which sensory input is progressively compressed and reconfigured along the cortical hierarchy according to task context.

In conclusion, the current study demonstrates that summary statistics are encoded not only in the higher‐order PPC but are also already robustly represented in the early V1. These representations are modulated by both stimulus variability and task context, and are subsequently transformed into categorical formats in PPC. Ultimately, our findings underscore the brain's capacity to transform variable sensory input into stable statistical summaries and categories through layered, distributed, and task‐sensitive codes. Understanding these mechanisms may shed light on broader principles of abstract information processing in the brain.

## 4 Experimental Section

### 4.1 Animals

In this study, a total of age‐matched 17 and 14 male C57BL/6J wild‐type mice (The Jackson Laboratory, RRID: IMSR_JAX:000664, 11–30 weeks old) were used for motion categorization task and passive viewing condition, respectively (For V1 recordings, 8 mice for only during the task, 4 mice for only during passive viewing, and 2 mice for both conditions. For PPC recordings, 2 mice for only during the task, 3 mice for only during passive viewing, and 5 mice for both conditions). For mice participating in both task and passive conditions, they always participated in the passive condition first to prevent their exposure to the categorization task from influencing the passive condition. For animals that completed both tasks at the same site, the two conditions were treated as independent sessions. These four groups of mice with different sample sizes were separated by task and recording area. Thus, during the motion categorization task or passive viewing, only within‐subject statistical comparisons between homogeneous and heterogeneous trials were performed in V1 and PPC, respectively. They were maintained under controlled environmental conditions at approximately 22°C with 50% relative humidity, on a 12‐hour light‐dark cycle. Prior to surgery, the mice were group‐housed (4–5 per cage) with ad libitum access to food and water in accordance with the PHS Policy on Humane Care and Use of Laboratory Animals. After head bar fixation and lens implantation surgery, they were housed individually in separate cages. During behavioral training and thereafter, water intake was restricted to approximately 1 mL per day to maintain 80% of their baseline body weight. All experimental procedures in this study were approved by the Institutional Animal Care and Use Committee (IACUC) of the Institute for Basic Science (IBS, approval number: IBS‐2021‐014), Daejeon, South Korea and complied with the ARRIVE guidelines.

### 4.2 Surgical Procedures

Mice were anesthetized with isoflurane (5% for induction, 1%–2% for maintenance) and secured in a stereotaxic frame (Narishige, Japan). Body temperature was maintained at 37°C using a heating pad (DC temperature control system, FHC Inc., Bowdoin, ME, USA). To prevent ocular dryness during the surgery, an ophthalmic ointment was applied to the eyes. Prior to the scalp incision, local anesthesia (0.5% bupivacaine in saline) was administered for analgesia. The scalp and periosteum covering the dorsal surface of the skull were then removed, and the skull surface was abraded to enhance adhesion for subsequent fixation. A circular craniotomy (1 mm in diameter) was made over either the left primary visual cortex (V1; A/P: −3.34 mm, M/L: 2.53 mm) or the left posterior parietal cortex (PPC; A/P: −2 mm, M/L: 1.7 mm), taking care to leave the dura intact. A virus expressing GCaMP6f (pENN.AAV.CaMKII.GCaMP6f.WPRE.SV40; Addgene Cat. #100834‐AAV1; RRID: Addgene_100834) was injected into the target region (100–150 nL, 1 nL/s, 200 mm deep from the dura) using glass pipettes connected to a hydraulic injection system (Nanoject III, Drummond Scientific, Broomall, PA, USA). The glass pipette was left in place for 10 min to allow for virus diffusion. The craniotomy was then sealed with bone wax, and the surgical incision was sutured. Three days after the virus injection, a GRIN lens (1 mm in diameter, Inscoix, CA, USA) was implanted above the virus injection site. The GRIN lens and a head bar were fixed to the skull using light‐cured dental composite and dental acrylic. At the end of the surgery, Ketoprofen (3 mL/kg) was administered intraperitoneally for analgesia and to reduce inflammation, with additional doses provided during the recovery period. After at least three weeks of recovery, a Miniscope baseplate was attached to the head using dental acrylic. After the last calcium imaging session, the mice were transcardially perfused with 4% paraformaldehyde (PFA) (T&I biotechnology, South Korea). Then, the brains were post‐fixed in 4% PFA for 24 h and cryo‐protected in 30% sucrose at 4°C for 48—72 h. Then, coronal sections (40 µm thick) were acquired using a cryostat microtome (CM 1950, Leica) and mounted on slides. GCaMP6f fluorescence was imaged using a slide scanner (Axio Scan Z1, Carl Zeiss, Germany) to verify the location of lens implantation and the expression of GCaMP6f.

### 4.3 Behavioral Apparatus

The experimental apparatus was built inside a soundproof box measuring 64 cm (W) × 64 cm (L) × 60 cm (H). Visual stimuli were presented on a 21‐inch LCD display (DELL, UltraSharp U2417H, 1920  ×  1080 resolution, 60 Hz refresh rate) positioned 15 cm away from the mouse. A head‐fixation device was mounted at the center of the box to secure the subject mouse in place. A decision wheel (3.00″ Precision Disk Wheel, Servocity 595720) was positioned to align with the mouse's forelimbs. An angular encoder (US Digital, E2‐32‐250‐NE‐H‐3‐B) was used to detect the direction and magnitude of wheel rotation. A linear actuator (mightyZap, 12Lf‐12PT‐27) was used to lock or release the wheel by extending or retracting its arm. A lickport, made from a stainless‐steel tube (1.5‐mm outer diameter, 1‐mm inner diameter) was placed in front of the mouse. Water rewards were delivered via a tube connecting the lickport to a water reservoir (a 10 mL syringe) placed 50 cm above the lickport, using gravity. A solenoid valve (161T011, NResearch Inc., NJ, USA) controlled the delivery of rewards. An Arduino board (Arduino UNO) was used to control the solenoid valve and linear actuator, and to read signals from the angular encoder. A custom graphic user interface, written with MATLAB (2019a, MathWorks, USA), was used to control task parameters and display task progress. The MATLAB support package for arduino hardware was used to control the Arduino board via a MATLAB script. A monochrome camera (PointGrey BFLY‐U3‐03S2M‐CS, Blackfly, Canada) was positioned on the side of the subject mice for monitoring licking behavior.

### 4.4 Stimulus Design

Visual stimuli were generated and presented using MATLAB and the Psychophysics Toolbox [70]. We employed unconventional versions of random dot kinematograms (RDKs). Five hundred dots (1.5° in diameter each) were randomly spawned within a circular area of 125° diameter on the screen. The dots moved at a speed of 200 pixels/s. When a dot reached the edge of the circle, it was repositioned to the diametrically opposite location of the circle while maintaining its motion direction. Each dot had a fixed lifetime of 0.5 s, after which it reappeared at a new random location within the stimulus area while maintaining the same motion direction. At stimulus onset, dots were initiated with different lifetimes to ensure a constant rate of dot reappearance throughout the stimulus presentation. In different trials, dots moved in either homogeneous or heterogeneous directions. In heterogeneous RDKs, each dot's motion direction was randomly drawn from a uniform distribution within one of three angular ranges: 90°, 180°, and 270°. The global mean motion direction of each RDK stimulus was one of eight directions, spaced at 45° intervals from 22.5° to 337.5°, relative to the rightward horizontal axis. These stochastic RDKs allowed for systematic variation in the range of the motion direction distribution while preserving the global mean motion direction. In homogeneous RDKs, all dots moved coherently in the same direction, selected from the same set of eight motion directions.

### 4.5 Behavioral Training Procedure

One day before the start of handling, the mice were placed on a water restriction schedule. After 2–3 days of handling and habituation to the head‐fixation device, the mice were head‐fixed and trained to spin the decision wheel to receive a water reward while viewing stationary dot stimuli for three days. Next, the mice were presented with homogeneous RDKs moving horizontally either to the left or right, and were trained to turn the wheel in the corresponding direction to receive a reward. In the next training phase, the mice learned to categorize four “easy” directions of homogeneous RDKs (directions close to horizontal directions, 22.5°, 157.5°, 202.5°, and 337.5° relative to the rightward horizontal direction) into left or right categories. Finally, the mice were trained to classify eight directions of homogeneous RDKs into left or right categories. These included the four easy directions and four additional “hard” directions (closer to the vertical category boundary, 67.5°, 112.5°, 247.5°, and 292.5° from the rightward horizontal direction). Mice advanced to the next training stage once their performance exceeded 80% accuracy for three consecutive days in the current stage. Note that only homogeneous RDKs were used throughout the training period.

### 4.6 Global Mean Motion Direction Discrimination Task

After completing the training, the mice performed a task in which they categorized eight motion directions of both homogeneous and heterogeneous RDKs, while calcium signals were recorded from the V1 or PPC. The task was conducted over three recording sessions on separate days, each using a different range of motion direction variability: 90°, 180°, or 270°. In each session, there were sixteen trial types, consisting of eight mean motion directions and two motion direction variances (homogeneous and one of three heterogeneous ranges). Each session included 160 homogeneous trials (20 trials per direction) and 160 heterogeneous trials, presented in random order. After a 2‐second interval, the RDK stimulus was presented. Stimuli remained on the screen for at least 4 s. During the first 4 s, the wheel brake prevented wheel movement. After this period, the wheel brake was released, allowing the mouse to turn the wheel. On each trial, the head‐fixed mouse indicated whether the global mean motion direction of the RDK fell into the left or right category by turning the wheel accordingly to receive a water reward.

### 4.7 Passive Viewing of Motion Stimuli

In passive‐viewing sessions, calcium activity was recorded from V1 or PPC while mice passively viewed the RDK stimuli. The stimulus set included the same number of randomly intermixed homogeneous and heterogeneous trials as in the task sessions. All experimental conditions were identical to those used in the task sessions, except that no categorization or behavioral response was required. Throughout the entire passive‐viewing session, the wheel brake remained engaged, and a water reward was delivered after the 4‐second stimulus presentation on each trial.

### 4.8 In Vivo Calcium Imaging

We used a Miniscope [33] to image GCaMP6f fluorescence signals from layer 2/3 neurons in V1 and PPC. Raw grayscale images (608  ×  608 resolution, 3.2 µm/pixel, 30 Hz) were acquired using the Bonsai software package (https://github.com/jonnew/Bonsai.miniscope). The stimulus onset timestamp for each trial was recorded in MATLAB, while image frame timestamps from the Miniscope were recorded in Bonsai. MATLAB and Bonsai communicated via the UDP protocol, enabling synchronization between stimulus events and imaging data.

### 4.9 Preprocessing of Calcium Imaging Data

To eliminate movement artifacts, raw fluorescence image frames were motion‐corrected using the NoRMCorre algorithm [71]. Fluorescence signals from individual neurons were then extracted using the CNMF‐E algorithm [72]. For each neuron, slow baseline drifts in the fluorescence traces were removed by subtracting the eighth percentile value within a sliding window of 900 frames. The resulting traces were then smoothed using a Savitzky–Golay filter (order 3, frame length 11) to improve the signal‐to‐noise ratio. Next, calcium events were detected from the *dF/F* traces. To estimate a noise threshold, all negative *dF/F* values and their absolute values were concatenated as these represent neural activity‐independent fluctuations. The interquartile range of this fluctuation was then computed, and the noise threshold was set at three times the interquartile value. Then, calcium event traces were generated by retaining *dF/F* values above this threshold value and replacing all values below it with zero. Neurons were excluded from further analysis if the number of negatively deflected calcium events exceeded 5% of the number of positively deflected events [73].

### 4.10 Behavioral Analysis

Behavioral performance in task sessions was plotted as the percentage of trials in which the mice turned the wheel to the “right”, as a function of the mean motion direction. For each category (left or right), two “easy” trials types (directions far from the vertical category boundary) and two “hard” trial types (directions near the boundary) were grouped together, respectively. To compare behavioral performance between homogeneous and heterogeneous trials, each mouse's behavioral performance was fitted with a cumulative normal distribution function separately for the two conditions. Curve fitting was performed using the psignifit toolbox (https://github.com/wichmann‐lab/psignifit), which utilized a maximum‐likelihood method and the Weibull function to model the underlying shape of the psychometric curve [74]. Threshold was determined as the global motion direction change required to achieve performance one standard deviation from the average of the cumulative normal function. The slope of the psychometric function corresponds to the reciprocal of the standard deviation (*𝜎*) of the fitted normal function. All statistical comparisons between homogeneous and heterogeneous trials were conducted using the slope (1/*𝜎*) of the normal curves.

We also calculated *d′* as a complementary index of sensitivity, using the well‐established relationship between percent correct in a 2AFC task and *d′* [75]:

$$d^{\prime }=\sqrt{2}\times Z\left(p\right),$$

where *p* is the proportion of correct responses and Z(·) denotes the inverse cumulative distribution function of the standard normal distribution. d′ were calculated for each mouse individually and analyzed using a linear mixed‐effects model, with the interaction of stimulus type (homogeneous vs. heterogeneous), difficulty of global motion direction (easy vs. hard), and session (90°, 180°, and 270°) as a fixed effect and mouse identity as a random effect:

$$d^{\prime }∼\mathrm{stimulus}\;\mathrm{type}\times \mathrm{difficulty}\times \mathrm{session}+\left(1\left|\mathrm{mouse}\right.\right)$$

Post hoc analyses were conducted on estimated marginal means (EMMs) using the R package *emmeans*, with Tukey's multivariate‐t adjustment applied for multiple comparisons. Specifically, we tested the simple effect of condition (hetero − homo) within each difficulty × session and performed Tukey‐adjusted pairwise comparisons among sessions (90°, 180°, and 270°). With this analysis, we tested, for each session and separately for the easy‐ and hard‐direction conditions, whether d′ differed significantly between homogeneous and heterogeneous trials.

### 4.11 Mean Motion Direction Selectivity of Individual Neurons

Direction tuning curves for each neuron were generated by plotting the mean calcium responses during the 4‐second stimulus period across all 20 trials for each mean motion direction. These responses were plotted against the mean motion directions. Direction‐selective neurons were identified based on two criteria: the DSI and the slope of the tuning curve. The DSI was calculated as:

$$DSI=\left(R_{pref}-R_{antipref}\right)/R_{pref}$$

where *R_pref_* is the neuronal response to the preferred direction and *R_antipref_* is the response to the opposite direction. We calculated the slope of each neuron's zero‐centered tuning curve by fitting a linear slope across the direction from −180° to 0° after averaging neural responses of neuronal tuning curves that were equidistant from 0 (e.g., +45° and − 45°, +90° and −90°). Neurons were classified as direction‐selective if they had a DSI value greater than 0.4 [34, 35, 36] and a slope value exceeding the 95th percentile of slope values computed from trial‐shuffled datasets.

### 4.12 Correlation of Direction‐Selective Responses Between the Homogeneous and Heterogeneous Conditions

For each direction‐selective neuron identified under the homogeneous condition, mean neural responses to each of the eight motion directions were computed separately for the homogeneous and heterogeneous trials. These direction‐wide neural response vectors were then concatenated across neurons resulting in two population‐level response arrays—one for each condition. To assess the similarity of these response patterns, the Pearson correlation coefficient between these arrays was calculated. The same analysis was repeated using direction‐selective neurons identified under the heterogeneous condition.

### 4.13 Confusion Matrix of Peak Response Directions Between the Homogeneous and Heterogeneous Conditions

For each session type, an 8 by 8 confusion matrix was constructed using all recorded neurons, including even those not direction‐selective. Rows and columns of the matrix correspond to the eight motion directions under the homogeneous and heterogeneous conditions, respectively. Each element in the matrix represents the proportion of the neurons whose peak directions matched the corresponding pair of directions in the two conditions. To assess statistical significance, the observed proportions were compared against a null distribution generated from 5000 surrogate datasets, in which trial labels were randomly shuffled. A proportion was considered significant if it exceeded the 95th percentile of the surrogate distribution. Multiple comparisons were corrected using the FDR method.

### 4.14 Recovering Mean Motion Direction From Calcium Activity Patterns in V1 and PPC Using Population Response Curve (PRC)

To infer the mean motion direction from the spatially distributed pattern of the full calcium signals in V1 and PPC, we used a PRC [37, 38, 39] (Figure S8). The PRC represented the collective activity of a group of neurons in response to a fixed global motion direction of RDK. Thus, when the response of each neuron (with different preferred global motion directions) to a single fixed global motion direction was plotted, the resulting curve across the population was the PRC. This was distinct from a population‐averaged tuning curve, which showed how a single neuron responded to varying global motion directions (Figure S8). The PRC instead fixed the global motion direction and examined responses across many neurons with different preferred global motion directions.

For each cell, we determined the tuning curve by averaging the 4 s post‐stimulus signals onset and found its preferred global motion direction. Subsequently, we constructed the PRC as a function of global motion direction at each time point as follows: For a given RDK (the black arrow in Figure S8a), the responses of all cells that prefer the presented global motion direction were averaged across those cells and trials (the blue curve in Figure S8a), resulting in the value of the PRC at the presented global motion direction (the blue point in Figure S8a). In the same way, the values of the PRC at other global motion directions were obtained for a given RDK: the responses of cells that prefer the global motion direction other than the presented one were averaged across those cells and trials (the red and the green curves in Figure S8a), resulting in the value of the PRC at other global motion directions (the red and green points in Figure S8a). For each of the eight presented global motion directions, the PRC was zero‐centered relative to the presented global motion direction. This procedure was repeated for all time points in the stimulus epoch. Zero‐centered PRCs were then separated into homogeneous and heterogeneous conditions in V1 and PPC.

To summarize the slope of the PRC as a function of time, we calculated the linear slope of the zero‐centered tuning curve from −180° to 0° at each time point in the epoch [53]. We averaged the zero‐centered PRCs that were equidistant from 0° (i.e., +45° and −45°, +90°, and −90°). We then fit a linear slope across the direction channels from −180° to 0°, separately for each time point, variance condition, and mouse. PRC slope was evaluated using one‐sample *t*‐tests (against 0). The difference in PRC slope between homogeneous and heterogeneous conditions was evaluated using two‐sample *t*‐tests. In doing so, a zero PRC slope corresponded to no global motion direction selectivity, while a higher PRC slope corresponded to greater global motion direction selectivity. Multiple comparisons across time points were corrected using non‐parametric cluster‐based permutation testing [40] (5000 permutations).

### 4.15 Quantification of Neural Perceptual Bias

Neural response bias toward the category center was quantified as follows. For each stimulus direction, the PRC was averaged over the 4‐second stimulus window. The resulting mean response curve was then fitted with a wrapped normal distribution, defined as:

$$f\left(\theta \right)=\sum_{k=-\infty }^{\infty }exp\left(-\frac{{\left(\theta -\mu +2\pi k\right)}^{2}}{2\sigma^{2}}\right)$$

where *µ* denotes the neuron's preferred direction (i.e., the peak of the tuning curve), *σ* is the tuning width, and *θ* is the stimulus direction.

Perceptual bias was defined as the angular difference between the fitted peak direction *(θ_fitted _*_peak_ = *µ*) and the actual presented stimulus direction (*θ*_sample_):

$$Perceptual\;bias=\theta_{fitted\;peak}-\theta_{sample}$$

A positive bias value indicates a response shifted to the category center relative to the stimulus direction, while a negative value indicates a response shifted to the category boundary.

The statistical significance of the bias values was assessed using one‐sample *t‐*tests, with FDR correction applied for multiple comparisons.

### 4.16 Representational Similarity Analysis (RSA)

For each time point, we trained linear discriminant analysis classifiers on population response patterns across neurons to discriminate between all possible pairs of global motion directions of homogeneous and heterogeneous RDKs (16 × 16 pairs of comparisons in total for each task and passive session). Trials in each task session were randomly divided into four independent chunks for the classification analysis. A fourfold cross‐validation procedure was used, with balanced trial numbers for each condition. To reduce trial‐to‐trial noise, new synthetic trials were created by randomly averaging 25% of trials within each chunk, repeated 500 times per condition. This yielded time‐resolved decoding accuracy for each pair of directions, forming 16 × 16 neural representational dissimilarity matrices (RDMs), where higher accuracy indicates greater neural dissimilarity.

Three model RDMs were constructed to test hypotheses about the structure of neural representations: (1) mean motion direction RDM, based on channel tuning functions for the eight directions; (2) variance RDM, indicating whether trial pairs shared the same motion direction variance; and (3) category RDM, indicating whether trial pairs belonged to the same directional category (left vs. right). Elements were assigned values from 0 (similar) to 1 (dissimilar) depending on model criteria. For each time point, a multiple linear regression was performed:

$$\begin{array}{ccc} RDM_{neuron} & = & \beta_{0}+\beta_{1}\times RDM_{mean}+\beta_{2}\times RDM_{variance}\\ & & +\;\beta_{3}\times RDM_{category} \end{array}$$

All model RDMs were z‐scored prior to regression. Beta coefficients were estimated per mouse and tested at the group level using cluster‐based permutation tests [40], to identify significant time windows.

### 4.17 MVPAs Separately Tracking Mean, Variance, and Category Information to Complement RSA

To address the issue of the collinearity among three model RDMs, we used time‐generalized multivariate pattern analyses to directly track the emergence of mean motion direction, motion variance, and category information over time in the calcium signal.

### 4.18 Recovering Mean Motion Direction Using an Inverted Encoding Model (IEM)

To reconstruct the mean motion direction from multivariate V1 and PPC activities, we used an inverted encoding model (IEM) [53] where each mean motion direction was represented using weights from a linear basis set of population tuning curves. Eight hypothetical channel tuning functions (CTFs) were centered at eight mean motion directions used in trials, evenly spaced from 22.5° to 337.5° in steps of 45°. Each basis function was a half‐sinusoidal function raised to the sixth power.

In each of three task sessions, the multivariate V1 or PPC activities of all the presented homogeneous RDKs labeled with their mean motion directions were trained and tested in the leave‐one‐trial‐out cross‐validation fashion. In detail, one trial was held out as the test set, and the decoder was trained on the remaining trials using the corresponding labels for the variable of interest and then used to predict the label of the held‐out trial. This procedure was repeated until every trial had served as the test set once, and accuracy was computed as the proportion of correctly classified trials. Importantly, this entire procedure was performed independently at each time point to obtain time‐resolved decoding performance. The multivariate V1 or PPC activities of heterogeneous trials were tested with the IEM weight matrix trained on the homogeneous trials. For each trial, the CTF was zero‐centered relative to the presented mean motion direction. This procedure was repeated for each time point in the epoch from −0.5 to 4 s after stimulus onset before moving to the next iteration. Zero‐centered mean motion direction‐selective tuning functions were separately averaged across trials for each homogeneous and heterogeneous trials (Figure S13a,b).

We constructed the IEM as:

$$B_{1}=WC_{1}$$

where *B*_1_is the training set (number of neurons × training set of trials) and *C*_1_is the hypothetical channel tuning functions (8 mean motion directions × training set of trials). We then estimated the weight matrix *W*(number of neurons × 8 mean motion directions) by multiplying both sides with the pseudoinverse of *C*_1_ as in the ordinary least squares (OLS):

$$\hat{W}=B_{1}C_{1}^{⊤}{\left(C_{1}C_{1}^{⊤}\right)}^{-1}$$

Using the estimated weight $\hat{W}$ and the test set *B*_2_(number of neurons × test set of trials), we estimated the population mean motion direction response ${\hat{C}}_{2}$ (8 directions × test set of trials):

$${\hat{C}}_{2}={\left({\hat{W}}^{⊤}\hat{W}\right)}^{-1}{\hat{W}}^{⊤}B_{2}$$

where ${\hat{C}}_{2}$ denotes the tuning curve of the test set, $\hat{W}$ is the weight matrix, ${\hat{W}}^{⊤}$ its transpose, and ${\hat{W}}^{-1}$ its pseudoinverse. For each time point in the training set, we applied the estimated weights to the test set at the same time point, and then zero‐centered the resulting tuning curve ${\hat{C}}_{2}$ relative to the labeled mean motion direction. All eight aligned CTFs were averaged.

To summarize the decoding accuracy, we calculated the linear slope of the CTFs. We reversed the signs of the mean motion direction channels 45°, 90°, and 135° to become −45°, −90°, and −135°, respectively. We then fitted the linear regression with an intercept across the eight channel responses (i.e., −180°, −135°, −135°, −90°, −90°, −45°, −45°, and 0°) and obtained the linear slope of the CTFs. Tuning curve slope was evaluated using one‐sample *t*‐tests (against 0). In doing so, a zero tuning curve slope corresponded to no mean motion direction selectivity, while a higher tuning curve slope corresponded to greater mean motion direction selectivity. Multiple comparisons across time points were corrected using non‐parametric cluster‐based permutation testing [40] (5000 permutations).

### 4.19 Classifying Motion Variance Using Support Vector Machine (SVM)

In each of three 90°, 180°, and 270° test sessions, we investigated if population activity patterns reflected the motion direction variance by discriminating homogeneous and heterogeneous trials using an SVM (MATLAB *fitcsvm* function) in both V1 and PPC. At each time point, we trained and tested the homogeneous versus heterogeneous RDK classifier with leave‐one‐trial‐out cross‐validation (Figure S14a,b). We used a non‐parametric cluster‐based permutation test [40] using one‐sample *t*‐tests to assess the time points where homogeneous and heterogeneous trials were significantly discriminated above the chance level of 0.5.

### 4.20 Classifying Motion Category Using Support Vector Machine (SVM)

For homogeneous, 90°‐, 180°‐, and 270°‐heterogeneous trials, we separately examined if population activity patterns discriminated the left and the right motion category using an SVM in both V1 and PPC (Figure S15a,b). At each time point of homogeneous trials, we trained and tested the left versus right classifier with leave‐one‐trial‐out cross‐validation, whereas we tested heterogeneous trials with the SVM decoder trained on homogeneous trials. We used non‐parametric cluster‐based permutation test [40] using one‐sample *t*‐tests to assess the time points where left and right motion category trials were significantly discriminated above the chance level of 0.5.

As significant mean direction encoding accuracy and category discrimination accuracy appeared in both V1 and PPC (Figures S13 and S15), we investigated which of the two was dominantly reflected in each brain area by comparing the decoding accuracy of within‐category mean motion direction and that of between‐category mean motion direction [28]. Specifically, from eight mean motion directions, we chose four pairs of mean directions that were 90° apart and discriminated each pair of mean directions using a binary SVM with repeated stratified fivefold cross‐validation (Figure 6d). Within‐category discrimination (WCD) was performed across the four pairs of mean directions that were 90° apart and stayed within the same category (1 vs. 3, 2 vs.7, 5 vs. 7 and 6 vs. 8). Between‐category discrimination (BCD) was performed across the four pairs of mean directions that were 90° apart and crossed the category boundary (1 vs. 7, 2 vs. 8, 3 vs. 5 and 4 vs. 6). We partitioned the entire homogeneous trials into five folds in a stratified manner so that the class proportions were preserved as closely as possible within each fold. In each repetition, we trained the SVM on four folds and evaluated it on the remaining fold, iterating through all five folds to complete one fivefold cross‐validation cycle. These decoders trained on homogeneous trials were applied to heterogeneous trials. To mitigate chance variability arising from a single data split, we repeated this fivefold procedure 100 times using different random stratified partitions. Both WCD and BCD accuracies were calculated separately for homogeneous, 90°‐, 180°‐, and 270°‐heterogeneous trials. We then averaged the WDC accuracies and BCD accuracies across four levels of motion variance and used them to evaluate the difference between WCD and BCD in V1 and PPC (Figure 6e,f). We used a non‐parametric cluster‐based permutation test [40] using one‐sample *t*‐tests to assess the time points where WCD (blue lines in Figure 6e,f) and BCD accuracies (red lines in Figure 6e,f) were significantly higher above the chance level of 0.5. We also used the same non‐parametric cluster‐based permutation test [40] using one‐sample *t*‐tests to assess the time points where BCD accuracies were significantly higher than WCD accuracies (black lines in Figure 6e,f).

### 4.21 Temporal Generalization (TG) Analysis

To assess when each of the mean, variance, and category information emerged as well as how stable it was over time, we implemented temporal generalization (TG) of the IEM and the binary SVM classifiers. Specifically, at a particular time point *t*, the IEM weight matrix or SVM classifiers trained at a particular time point were generalized to the test set at time *t*′. We iterated this procedure until the IEM or SVM classifier trained at every time point was generalized to the calcium signals obtained at every time point, making a two‐dimensional TG matrix of tuning curve slopes or decoding accuracies (c and d in Figures S13–S15), which showed only TG maps of three heterogeneous trials tested on IEM or SVM trained on homogeneous trials. Multiple comparisons across all train–test time point pairs were corrected using a nonparametric cluster‐based permutation test [40] (5000 permutations). Train–test pairs with decoding performance significantly above chance (one‐sample *t*‐test) were identified as significant temporal generalization regions.

### 4.22 Statistical Analysis

All statistical analyses were performed using MATLAB (R2020a, MathWorks) and R (version 4.3.3). Data were reported as mean ± standard error of the mean (SEM). The exact sample size (*n*) for each analysis is provided in the corresponding figure legends and supporting tables. Depending on the type of analysis, we used parametric or non‐parametric tests as appropriate, including *t*‐tests, analysis of variance (ANOVA), linear mixed‐effects models, correlation analyses, and non‐parametric cluster‐based permutation tests for time‐resolved data. All statistical tests were explicitly performed as one‐sided or two‐sided as needed, and the significance level (*α*) was set to 0.05. Where necessary, *p*‐values were adjusted for multiple comparisons (Tukey's multiple comparison test or FDR correction), and specific details are described in the corresponding figure legends and supporting tables. Asterisks indicate statistical significance and are denoted as follows: n.s., not significant; *p < 0.05; **p < 0.01; ***p < 0.001; ****p < 0.0001.

## Author Contributions

Conceptualization: Y.B.L., D.L., and Y.J.K. Methodology: Y.B.L., O.J., G.E.J., D.L., and Y.J.K. Investigation: Y.B.L., O.J., G.E.J., D.L., and Y.J.K. Visualization: Y.B.L., O.J., D.L., and Y.J.K. Supervision: D.L. and Y.J.K. Writing the original draft: Y.B.L., D.L., and Y.J.K. Writing the review and editing: Y.B.L., D.L., and Y.J.K.

## Funding

This work was supported by the Institute of Basic Science (IBS‐R001‐D2).

## Conflicts of Interest

The authors declare no conflicts of interest.

## Supporting information




**Supporting File 1**: advs74706‐sup‐0001‐SuppMat.docx.

**Supporting File 2**: advs74706‐sup‐0002‐Movie S1.mp4.

**Supporting File 3**: advs74706‐sup‐0003‐Movie S2.mp4.

**Supporting File 4**: advs74706‐sup‐0004‐Movie S3.mp4.

## Data Availability

The data that support the findings of this study are available from the corresponding author upon reasonable request.;

## References

[1] S. Dakin , The Oxford Handbook of Perceptual Organization, ed. J. Wagemans , (Oxford University Press, 2015).

[2] D. Marr , Vision: A Computational Investigation Into the human Representation and Processing of Visual Information (MIT Press, 2010), 429, 10.7551/mitpress/9780262514620.001.0001.

[3] D. Whitney and A. Y. Leib , “Ensemble Perception,” Annual Review of Psychology 69 (2018): 105–129, 10.1146/annurev-psych-010416-044232. 28892638

[4] G. A. Alvarez , “Representing Multiple Objects as an Ensemble Enhances Visual Cognition,” Trends in Cognitive Sciences 15 (2011): 122–131, 10.1016/j.tics.2011.01.003. 21292539

[5] S. C. Chong and A. Treisman , “Statistical Processing: Computing the Average Size in Perceptual Groups,” Vision Research 45 (2005): 891–900, 10.1016/j.visres.2004.10.004. 15644229

[6] J. E. Corbett , I. Utochkin , and S. Hochstein , The Pervasiveness of Ensemble Perception: Not Just Your Average Review (Cambridge University Press, 2023), 10.1017/9781009222716.

[7] D. Ariely , “Seeing Sets: Representation by Statistical Properties,” Psychological Science 12 (2001): 157–162, 10.1111/1467-9280.00327. 11340926

[8] D. Whitney , J. Haberman , and T. D. Sweeny , “From Textures to Crowds: Multiple Levels of Summary Statistical Perception,” in The New Visual Neurosciences, ed. J. S. Werner and L. M. Chalupa (MIT Press, 2014), 695–710.

[9] M. A. Cohen , D. C. Dennett , and N. Kanwisher , “What Is the Bandwidth of Perceptual Experience?,” Trends in Cognitive Sciences 20 (2016): 324–335, 10.1016/j.tics.2016.03.006. 27105668 PMC4898652

[10] F. Campana and C. Tallon‐Baudry , “Anchoring Visual Subjective Experience in a Neural Model: the Coarse Vividness Hypothesis,” Neuropsychologia 51 (2013): 1050–1060, 10.1016/j.neuropsychologia.2013.02.021. 23499720

[11] N. Khayat and S. Hochstein , “Relating Categorization to Set Summary Statistics Perception,” Attention, Perception, & Psychophysics 81 (2019): 2850–2872, 10.3758/s13414-019-01792-7. PMC6856046 31243687

[12] I. S. Utochkin , “Ensemble Summary Statistics as a Basis for Rapid Visual Categorization,” Journal of Vision 15 (2015): 8, 10.1167/15.4.8. 26317396

[13] D. E. Stansbury , T. Naselaris , and J. L. Gallant , “Natural Scene Statistics Account for the Representation of Scene Categories in Human Visual Cortex,” Neuron 79 (2013): 1025–1034, 10.1016/j.neuron.2013.06.034. 23932491 PMC5464350

[14] A. Oliva and A. Torralba , “Building the Gist of a Scene: the Role of Global Image Features in Recognition,” Progress in Brain Research 155 (2006): 23–36. 17027377 10.1016/S0079-6123(06)55002-2

[15] F. Rigoli , G. Pezzulo , R. Dolan , and K. Friston , “A Goal‐Directed Bayesian Framework for Categorization,” Frontiers in Psychology 8 (2017): 408, 10.3389/fpsyg.2017.00408. 28382008 PMC5360703

[16] A. Oliva and A. Torralba , “The Role of Context in Object Recognition,” Trends in Cognitive Sciences 11 (2007): 520–527, 10.1016/j.tics.2007.09.009. 18024143

[17] N. Khayat , S. Fusi , and S. Hochstein , “Perceiving Ensemble Statistics of Novel Image Sets,” Attention, Perception, & Psychophysics 83 (2021): 1312–1328, 10.3758/s13414-020-02174-0. PMC8049939 33420715

[18] S. Hochstein and M. Ahissar , “View From the Top,” Neuron 36 (2002): 791–804, 10.1016/S0896-6273(02)01091-7. 12467584

[19] I. S. Utochkin , J. Choi , and S. C. Chong , “A Population Response Model of Ensemble Perception,” Psychological Review 131 (2024): 36–57, 10.1037/rev0000426. 37011150

[20] M. M. Robinson and T. F. Brady , “A Quantitative Model of Ensemble Perception as Summed Activation in Feature Space,” Nature Human Behaviour 7 (2023): 1638–1651, 10.1038/s41562-023-01602-z. PMC10810262 37402880

[21] B. S. Webb , T. Ledgeway , and P. V. McGraw , “Cortical Pooling Algorithms for Judging Global Motion Direction,” Proceedings of the National Academy of Sciences 104 (2007): 3532–3537, 10.1073/pnas.0611288104. PMC1805614 17360678

[22] B. S. Webb , T. Ledgeway , and P. V. McGraw , “Relating Spatial and Temporal Orientation Pooling to Population Decoding Solutions in Human Vision,” Vision Research 50 (2010): 2274–2283, 10.1016/j.visres.2010.04.019. 20447413 PMC2982753

[23] J. Freeman and E. P. Simoncelli , “Metamers of the Ventral Stream,” Nature Neuroscience 14 (2011): 1195–1201. 21841776 10.1038/nn.2889 PMC3164938

[24] S. Treue , K. Hol , and H.‐J. Rauber , “Seeing Multiple Directions of Motion—Physiology and Psychophysics,” Nature Neuroscience 3 (2000): 270–276, 10.1038/72985. 10700260

[25] W. Chaney , J. Fischer , and D. Whitney , “The Hierarchical Sparse Selection Model of Visual Crowding,” Frontiers in Integrative Neuroscience 8 (2014): 73, 10.3389/fnint.2014.00073. 25309360 PMC4174752

[26] D. W. Williams and R. Sekuler , “Coherent Global Motion Percepts From Stochastic Local Motions,” Vision Research 24 (1984): 55–62, 10.1016/0042-6989(84)90144-5. 6695508

[27] T. Marques , M. T. Summers , G. Fioreze , et al., “A Role for Mouse Primary Visual Cortex in Motion Perception,” Current Biology 28 (2018): 1703–1713, 10.1016/j.cub.2018.04.012. 29779878 PMC5988967

[28] D. J. Freedman and J. A. Assad , “Experience‐Dependent Representation of Visual Categories in Parietal Cortex,” Nature 443 (2006): 85–88, 10.1038/nature05078. 16936716

[29] J. K. Fitzgerald , D. J. Freedman , and J. A. Assad , “Generalized Associative Representations in Parietal Cortex,” Nature Neuroscience 14 (2011): 1075–1079, 10.1038/nn.2878. 21765425 PMC3145031

[30] L. Zhong , Y. Zhang , C. A. Duan , J. Deng , J. Pan , and N.‐L. Xu , “Causal Contributions of Parietal Cortex to Perceptual Decision‐Making During Stimulus Categorization,” Nature Neuroscience 22 (2019): 963–973, 10.1038/s41593-019-0383-6. 31036942

[31] D. J. Freedman and J. A. Assad , “A Proposed Common Neural Mechanism for Categorization and Perceptual Decisions,” Nature Neuroscience 14 (2011): 143–146, 10.1038/nn.2740. 21270782

[32] D. J. Cai , D. Aharoni , T. Shuman , et al., “A Shared Neural Ensemble Links Distinct Contextual Memories Encoded Close in Time,” Nature 534 (2016): 115–118, 10.1038/nature17955. 27251287 PMC5063500

[33] D. Aharoni and T. M. Hoogland , “Circuit Investigations With Open‐Source Miniaturized Microscopes: Past , Present and Future,” Frontiers in Cellular Neuroscience 13 (2019): 141, 10.3389/fncel.2019.00141. 31024265 PMC6461004

[34] T. L. Ribeiro , P. Jendrichovsky , S. Yu , et al., “Trial‐by‐Trial Variability in Cortical Responses Exhibits Scaling of Spatial Correlations Predicted From Critical Dynamics,” Cell Reports 43 (2024): 113762, 10.1016/j.celrep.2024.113762. 38341856 PMC10956720

[35] H. Wang , O. Dey , W. N. Lagos , et al., “Parallel Pathways Carrying Direction‐and Orientation‐Selective Retinal Signals to Layer 4 of the Mouse Visual Cortex,” Cell Reports 43 (2024): 113830. 38386556 10.1016/j.celrep.2024.113830 PMC11111173

[36] T. Yokoyama , S. Manita , H. Uwamori , et al., “A Multicolor Suite for Deciphering Population Coding of Calcium and cAMP In Vivo,” Nature Methods 21 (2024): 897–907, 10.1038/s41592-024-02222-9. 38514778 PMC11093745

[37] P. Dayan and L. F. Abbott , Theoretical Neuroscience: Computational and Mathematical Modeling of Neural Systems (MIT Press, 2005).

[38] N. Qian , M. E. Goldberg , and M. Zhang , “Tuning Curves vs. population Responses, and Perceptual Consequences of Receptive‐Field Remapping,” Frontiers in Computational Neuroscience 16 (2023): 1060757, 10.3389/fncom.2022.1060757. 36714528 PMC9880053

[39] C. Papadimitriou , R. L. White , and L. H. Snyder , “Ghosts in the Machine II: Neural Correlates of Memory Interference From the Previous Trial,” Cerebral Cortex 27 (2017): 2513–2527. 27114176 10.1093/cercor/bhw106 PMC6059123

[40] E. Maris and R. Oostenveld , “Nonparametric Statistical Testing of EEG‐ and MEG‐Data,” Journal of Neuroscience Methods 164 (2007): 177–190, 10.1016/j.jneumeth.2007.03.024. 17517438

[41] E. F. Ester , T. C. Sprague , and J. T. Serences , “Categorical Biases in Human Occipitoparietal Cortex,” The Journal of Neuroscience 40 (2020): 917–931, 10.1523/JNEUROSCI.2700-19.2019. 31862856 PMC6975303

[42] Y. Xin , L. Zhong , Y. Zhang , T. Zhou , J. Pan , and N.‐L. Xu , “Sensory‐to‐Category Transformation via Dynamic Reorganization of Ensemble Structures in Mouse Auditory Cortex,” Neuron 103 (2019): 909–921, 10.1016/j.neuron.2019.06.004. 31296412

[43] N. Kriegeskorte , M. Mur , and P. Bandettini , “Representational Similarity Analysis—Connecting the Branches of Systems Neuroscience,” Frontiers in Systems Neuroscience 2 (2008): 4. 19104670 10.3389/neuro.06.004.2008 PMC2605405

[44] H. Nili , C. Wingfield , A. Walther , L. Su , W. Marslen‐Wilson , and N. Kriegeskorte , “A Toolbox for Representational Similarity Analysis,” PLOS Computational Biology 10 (2014): 1003553, 10.1371/journal.pcbi.1003553. PMC3990488 24743308

[45] A. S. Morcos and C. D. Harvey , “History‐Dependent Variability in Population Dynamics During Evidence Accumulation in Cortex,” Nature Neuroscience 19 (2016): 1672–1681, 10.1038/nn.4403. 27694990 PMC5127723

[46] L. N. Driscoll , N. L. Pettit , M. Minderer , S. N. Chettih , and C. D. Harvey , “Dynamic Reorganization of Neuronal Activity Patterns in Parietal Cortex,” Cell 170 (2017): 986–999, 10.1016/j.cell.2017.07.021. 28823559 PMC5718200

[47] A. Akrami , C. D. Kopec , M. E. Diamond , and C. D. Brody , “Posterior Parietal Cortex Represents Sensory History and Mediates Its Effects on Behaviour,” Nature 554 (2018): 368–372, 10.1038/nature25510. 29414944

[48] J.‐R. King and S. Dehaene , “Characterizing the Dynamics of Mental Representations: The Temporal Generalization Method,” Trends in Cognitive Sciences 18 (2014): 203–210, 10.1016/j.tics.2014.01.002. 24593982 PMC5635958

[49] G. Palagina , J. F. Meyer , and S. M. Smirnakis , “Complex Visual Motion Representation in Mouse Area V1,” The Journal of Neuroscience 37 (2017): 164–183, 10.1523/JNEUROSCI.0997-16.2017. 28053039 PMC5214628

[50] C.‐J. Jeon , E. Strettoi , and R. H. Masland , “The Major Cell Populations of the Mouse Retina,” The Journal of Neuroscience 18 (1998): 8936–8946, 10.1523/JNEUROSCI.18-21-08936.1998. 9786999 PMC6793518

[51] M. Rivlin‐Etzion , K. Zhou , W. Wei , et al., “Transgenic Mice Reveal Unexpected Diversity of On‐off Direction‐Selective Retinal Ganglion Cell Subtypes and Brain Structures Involved in Motion Processing,” Journal of Neuroscience 31 (2011): 8760–8769, 10.1523/JNEUROSCI.0564-11.2011. 21677160 PMC3139540

[52] C. M. Niell and M. P. Stryker , “Highly Selective Receptive Fields in Mouse Visual Cortex,” The Journal of Neuroscience 28 (2008): 7520–7536, 10.1523/JNEUROSCI.0623-08.2008. 18650330 PMC3040721

[53] J. Do , K. Y. Eo , O. James , J. Lee , and Y.‐J. Kim , “The Representational Dynamics of Sequential Perceptual Averaging,” The Journal of Neuroscience 42 (2022): 1141–1153, 10.1523/JNEUROSCI.0628-21.2021. 34903571 PMC8824498

[54] K.‐J. Tark , M.‐S. Kang , S. C. Chong , and W. M. Shim , “Neural Representations of Ensemble Coding in the Occipital and Parietal Cortices,” NeuroImage 245 (2021): 118680. 34718139 10.1016/j.neuroimage.2021.118680

[55] H. Y. Im , D. N. Albohn , T. G. Steiner , C. A. Cushing , R. B. Adams , and K. Kveraga , “Differential Hemispheric and Visual Stream Contributions to Ensemble Coding of Crowd Emotion,” Nature Human Behaviour 1 (2017): 828–842, 10.1038/s41562-017-0225-z. PMC5716353 29226255

[56] R. Desimone and S. J. Schein , “Visual Properties of Neurons in Area V4 of the Macaque: Sensitivity to Stimulus Form,” Journal of Neurophysiology 57 (1987): 835–868, 10.1152/jn.1987.57.3.835. 3559704

[57] J. S. Cant and Y. Xu , “Object Ensemble Processing in human Anterior‐Medial Ventral Visual Cortex,” The Journal of Neuroscience 32 (2012): 7685–7700, 10.1523/JNEUROSCI.3325-11.2012. 22649247 PMC6703596

[58] M. M. Roth , F. Helmchen , and B. M. Kampa , “Distinct Functional Properties of Primary and Posteromedial Visual Area of Mouse Neocortex,” Journal of Neuroscience 32 (2012): 9716–9726, 10.1523/JNEUROSCI.0110-12.2012. 22787057 PMC6622284

[59] C. D. Harvey , P. Coen , and D. W. Tank , “Choice‐Specific Sequences in Parietal Cortex During a Virtual‐Navigation Decision Task,” Nature 484 (2012): 62–68, 10.1038/nature10918. 22419153 PMC3321074

[60] T. D. Hanks , C. D. Kopec , B. W. Brunton , C. A. Duan , J. C. Erlich , and C. D. Brody , “Distinct Relationships of Parietal and Prefrontal Cortices to Evidence Accumulation,” Nature 520 (2015): 220–223, 10.1038/nature14066. 25600270 PMC4835184

[61] H. Shibasaki and M. Hallett , “What Is the Bereitschaftspotential?,” Clinical Neurophysiology 117 (2006): 2341–2356, 10.1016/j.clinph.2006.04.025. 16876476

[62] A. Funamizu , B. Kuhn , and K. Doya , “Neural Substrate of Dynamic Bayesian Inference in the Cerebral Cortex,” Nature Neuroscience 19 (2016): 1682–1689, 10.1038/nn.4390. 27643432

[63] A. R. Girshick , M. S. Landy , and E. P. Simoncelli , “Cardinal Rules: Visual Orientation Perception Reflects Knowledge of Environmental Statistics,” Nature Neuroscience 14 (2011): 926–932, 10.1038/nn.2831. 21642976 PMC3125404

[64] T. Yang and M. N. Shadlen , “Probabilistic Reasoning by Neurons,” Nature 447 (2007): 1075–1080, 10.1038/nature05852. 17546027

[65] L. N. Katz , J. L. Yates , J. W. Pillow , and A. C. Huk , “Dissociated Functional Significance of Decision‐Related Activity in the Primate Dorsal Stream,” Nature 535 (2016): 285–288, 10.1038/nature18617. 27376476 PMC4966283

[66] D. Lyamzin and A. Benucci , “The Mouse Posterior Parietal Cortex: Anatomy and Functions,” Neuroscience Research 140 (2019): 14–22, 10.1016/j.neures.2018.10.008. 30465783

[67] J. Zylberberg , “The Role of Untuned Neurons in Sensory Information Coding,” BioRxiv (2018): 134379, 10.1101/134379.

[68] M. Levy , O. Sporns , and J. N. MacLean , “Network Analysis of Murine Cortical Dynamics Implicates Untuned Neurons in Visual Stimulus Coding,” Cell Reports 31 (2020): 107483, 10.1016/j.celrep.2020.03.047. 32294431 PMC7218481

[69] R. J. Rabinovich , D. D. Kato , and R. M. Bruno , “Learning Enhances Encoding of Time and Temporal Surprise in Mouse Primary Sensory Cortex,” Nature Communications 13 (2022): 5504, 10.1038/s41467-022-33141-y. PMC9489862 36127340

[70] D. H. Brainard , “The Psychophysics Toolbox,” Spatial Vision 10 (1997): 433–436, 10.1163/156856897X00357. 9176952

[71] E. A. Pnevmatikakis and A. Giovannucci , “NoRMCorre: an Online Algorithm for Piecewise Rigid Motion Correction of Calcium Imaging Data,” Journal of Neuroscience Methods 291 (2017): 83–94, 10.1016/j.jneumeth.2017.07.031. 28782629

[72] P. Zhou , S. L. Resendez , J. Rodriguez‐Romaguera , et al., “Efficient and Accurate Extraction of In Vivo Calcium Signals From Microendoscopic Video Data,” eLife 7 (2018): 28728. 10.7554/eLife.28728 PMC5871355 29469809

[73] E. Kong , K.‐H. Lee , J. Do , P. Kim , and D. Lee , “Dynamic and Stable Hippocampal Representations of Social Identity and Reward Expectation Support Associative Social Memory in Male Mice,” Nature Communications 14 (2023): 2597, 10.1038/s41467-023-38338-3. PMC10163237 37147388

[74] F. A. Wichmann and N. J. Hill , “The Psychometric Function: I. fitting, Sampling, and Goodness of Fit,” Perception & Psychophysics 63 (2001): 1293–1313, 10.3758/BF03194544. 11800458

[75] D. M. Green and J. A. Swets , Signal Detection Theory and Psychophysics (APA PsycInfo, 1966).

