# Supplementary material for: Hierarchical Summary Statistics Encoding Across Primary Visual and Posterior Parietal Cortices
Source: Adv Sci (Weinh). 2026 Mar 22;13(27):e12369. doi: 10.1002/advs.202512369 (PMC13170198; doi:10.1002/advs.202512369)
Supplement: Supplementary file 1 — Supporting File 1: advs74706‐sup‐0001‐SuppMat.docx. [file ADVS-13-e12369-s002.docx]

**Supporting Information for**

**Hierarchical summary statistics encoding across primary visual and posterior parietal cortices**

Young-Beom Lee^a^, Oliver James^a^, Gaeun Jung^a,b^, Doyun Lee^a*^, Yee-Joon Kim^a*^

*Corresponding authors. Email: [leedoyun@ibs.re.kr](mailto:leedoyun@ibs.re.kr) & [joon@ibs.re.kr](mailto:joon@ibs.re.kr)

**This PDF file includes:**

Figures S1 to S15

Tables S1 to S5

Movies S1 to S3

**Other Supplementary Materials for this manuscript include the following:**

Movies S1 to S3


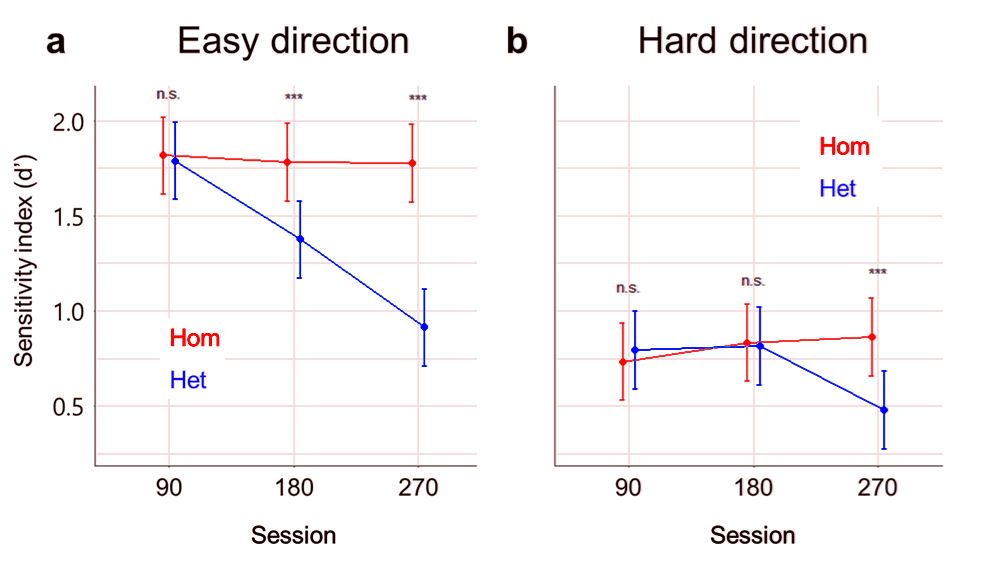


**Figure S1** **a, b** Comparison of category discrimination accuracy (d′) between homogeneous and heterogeneous trials for easy (a) and hard (b) type of trials. The x-axis shows three 90°, 180°, and 270° sessions and the y-axis shows d′. The red and blue traces indicate d′ for homogeneous and heterogeneous trials, respectively. In each session, d′ values for homogeneous and heterogeneous trials are compared. In the easy type of trials, d′ showed a significant difference between homogeneous and heterogeneous trials in the 180° and 270° sessions (180°: homo *d′* − hetero *d′* = 0.41, *t(176) =* 4.21*, p =* 4.08×10⁻⁵*, Cohen’s d =* 1.44. 270°: homo *d′* − hetero *d′* = 0.86, *t(176) =* 8.93*, p =* 5.64×10⁻¹⁶*, Cohen’s d =* 3.06), whereas in the hard type of trials, d′ was significantly different between homogeneous and heterogeneous trials only in the 270° session (homo *d′* − hetero *d′* = 0.38, *t(176) =* 3.96*, p =* 1.1×10⁻^4^*, Cohen’s d =* 3.06).

Data presented as the mean ± SEM, N = 17, p-values were calculated using R toolbox LMM (lme4/lmerTest) with fixed effects of stimulus type (Hom vs. Het), difficulty of global direction (hard directions vs easy directions), variance of session (90$^{\circ}$, 180$^{\circ}$, 270$^{\circ}$ session), and random intercepts for Mouse; two-tailed, Post hoc *emmeans* with Tukey adjustment, ***p < 0.001.


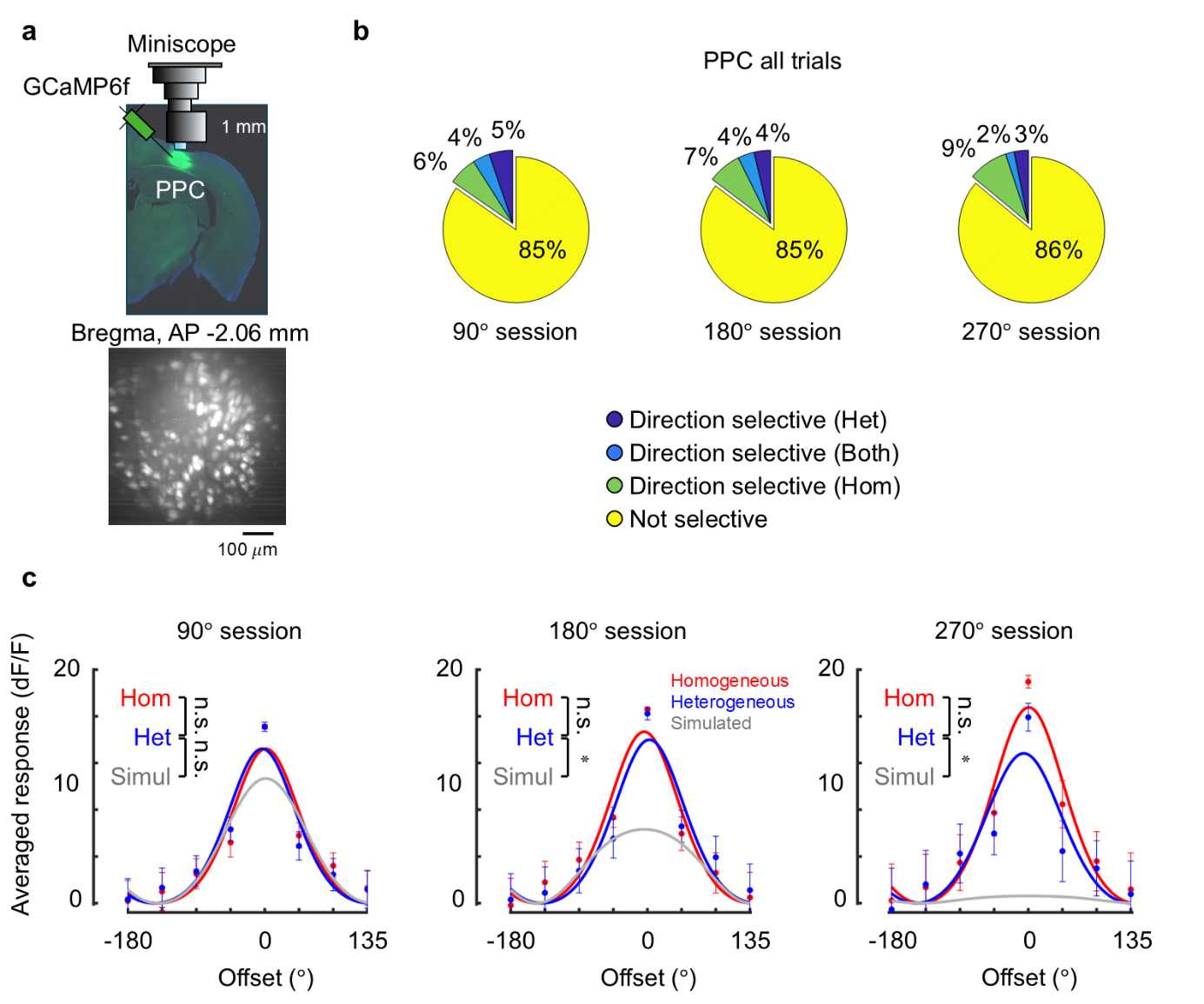


**Figure S1** Global motion direction-selective PPC neurons in response to homogeneous and heterogeneous RDKs during motion categorization task

**a** (Top) One-photon calcium imaging of L2/3 pyramidal PPC neurons expressing GCaMP6f. (Bottom) A frame-averaged image of PPC neurons of a trained animal.

**b** Proportions of global motion direction-selective PPC neurons in in 90°, 180°, and 270° sessions. Global motion direction-selective neurons were classified based on the direction selectivity index and the slope of single neuronal tuning curve. Of all PPC neurons that passed the DSI threshold of 0.4, only neurons with slopes steeper than the top 5% of the slope distribution calculated from the trial-shuffled dataset were considered as global motion direction-selective neurons (5000 permutations).

**c** Population-averaged tuning curves of global motion direction-selective PPC neurons in 90°, 180°, and 270° sessions. The red and blue curve indicate the tuning curve for homogeneous and heterogeneous RDKs, respectively. The gray curve is a simulated tuning curve for heterogeneous RDKs using the direction-selective neurons identified in the homogeneous trials. The simulation was conducted 1,000 times by resampling tuning curves of homogeneous RDKs, with the number of simulated neurons matched to the number of direction-selective neurons observed in the heterogeneous condition for each of three test sessions.

Data presented as mean ± SEM, 90° session (n_hom_ = 101 n_het_ = 90), 180° session (n_hom_ = 109 n_het_ = 72), 270° session (n_hom_ = 107 n_het_ = 51), p-values were calculated using Welch’s t-test. *p < 0.05.


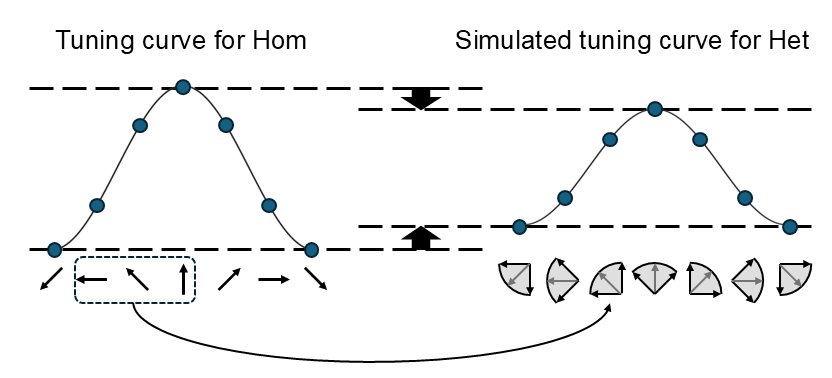


**Figure S2** Simulated tuning curve for heterogeneous condition with reduced gain, derived from tuning curves for homogeneous condition. Noisy input composed of a wider range of local motion directions, as in heterogeneous RDKs, is known to reduce the overall sensitivity or gain of neurons, thereby broadening their tuning curves. Based on this perspective, we assumed that the neural responses to a given range of motion directions in heterogeneous RDKs could be approximated by the average responses within that range on the tuning curves obtained from homogeneous trials. Using this assumption, we simulated tuning curves with reduced gain to model the neural responses to heterogeneous RDKs.


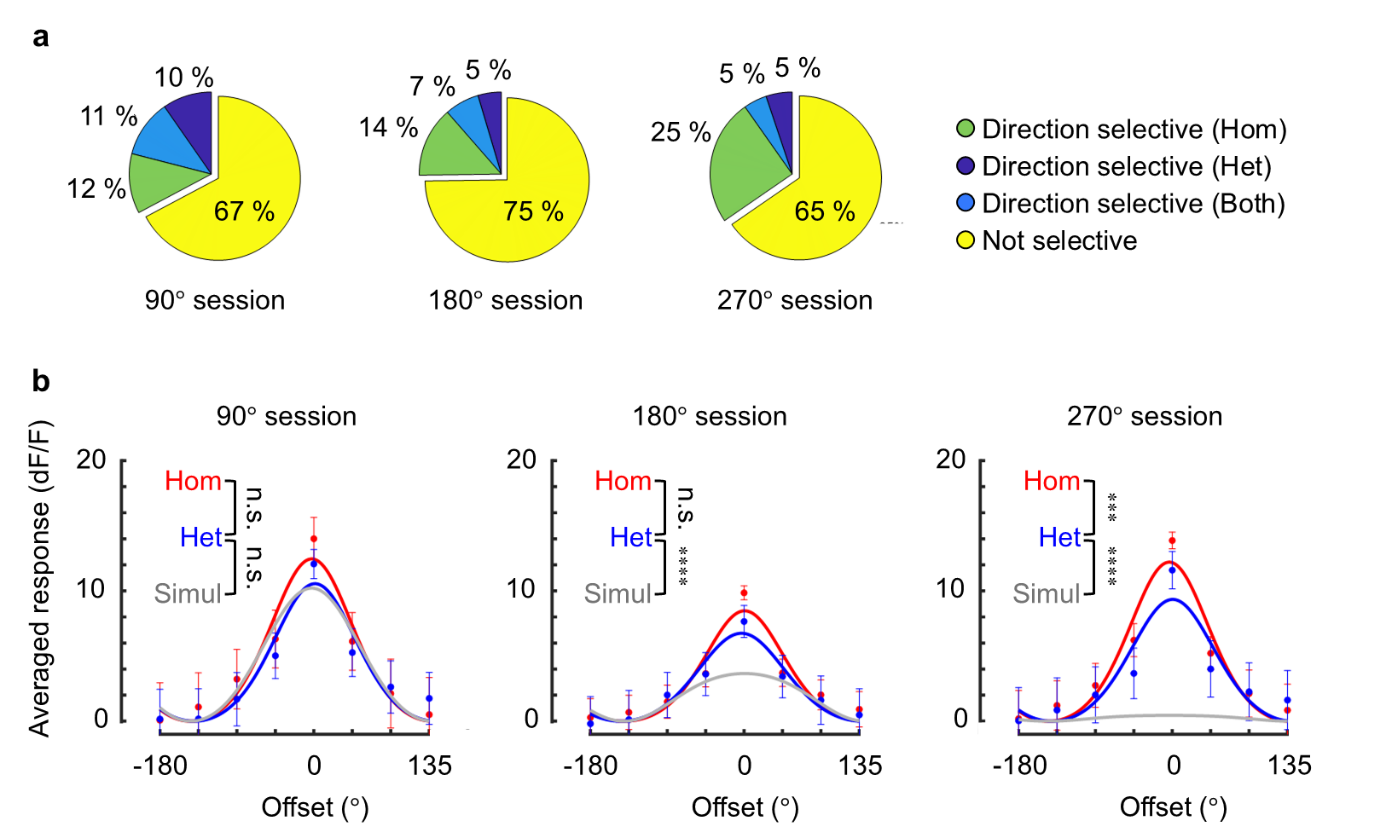


**Figure S3** Global motion direction-selective V1 neurons in response to homogeneous and heterogeneous RDKs during passive viewing

**a** Proportions of global motion direction-selective V1 neurons in 90°, 180°, and 270° sessions.

**b** Population-averaged tuning curves of global motion direction-selective V1 neurons in 90°, 180°, and 270° sessions.

Data presented as mean ± SEM, 90° session (n_hom_ = 98 n_het_ = 89), 180° session (n_hom_ = 139 n_het_ = 77), 270° session (n_hom_ = 154 n_het_ = 51), p-values were calculated using Welch’s t-test. ***p < 0.001, ****p < 0.0001.


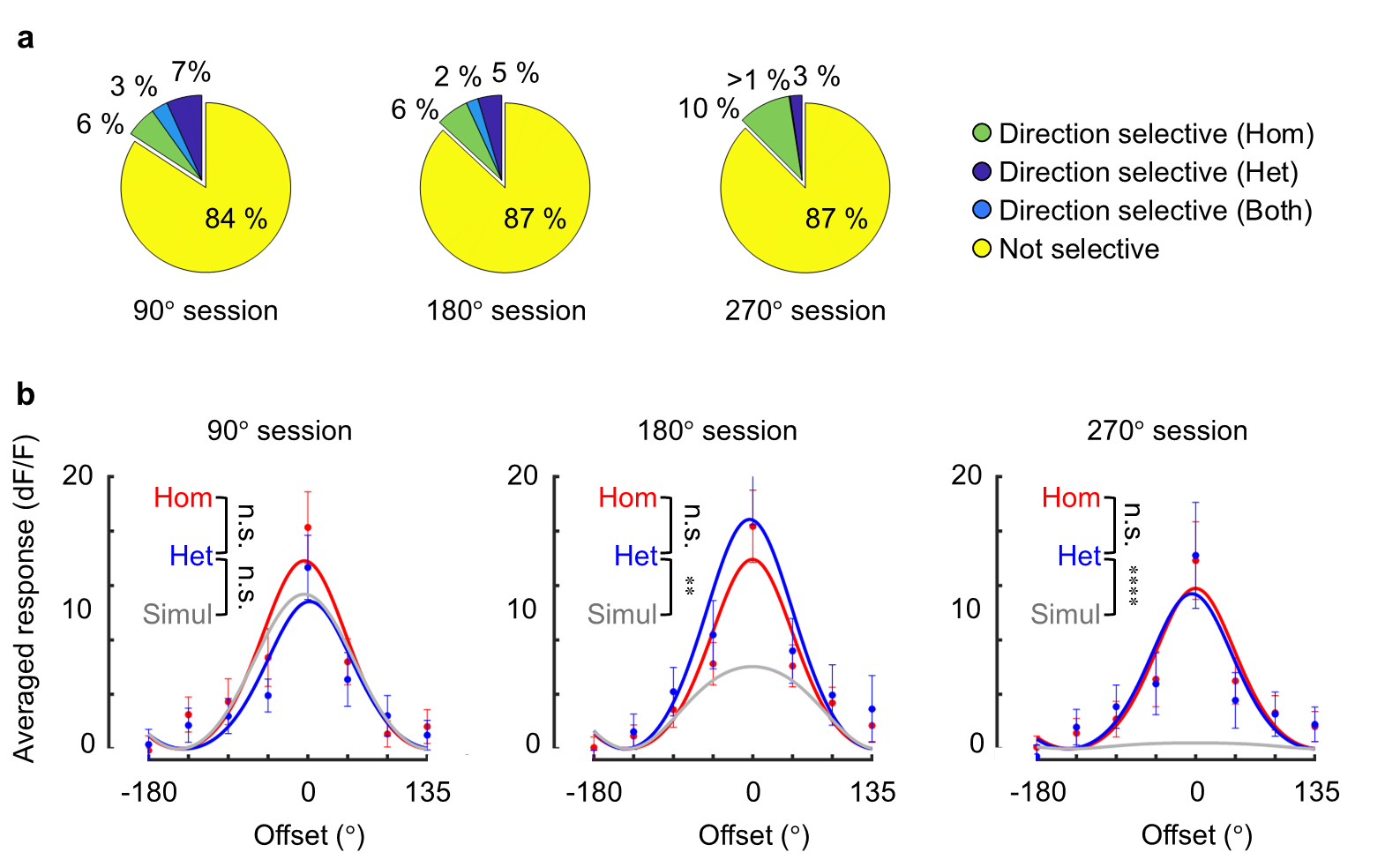


**Figure S4** Global motion direction-selective PPC neurons in response to homogeneous and heterogeneous RDKs during passive viewing

**a** Proportions of global motion direction-selective PPC neurons in 90°, 180°, and 270° sessions.

**b** Population-averaged tuning curves of global motion direction-selective PPC neurons in 90°, 180°, and 270° sessions.

Data presented as mean ± SEM, 90° session (n_hom_ = 95 n_het_ = 104), 180° session (n_hom_ = 96 n_het_ = 77), 270° session (n_hom_ = 103 n_het_ = 25), p-values were calculated using Welch’s t-test. **p < 0.01, ****p < 0.0001.

**Figure S6** Global motion direction-selective neurons in V1 and PPC vary between odd and even trials of the same homogeneous and heterogeneous RDK trials (related to Figure 3) during motion categorization task.

**a** Trials used for analysis in each condition

**b-c** Proportions of global motion direction-selective V1 neurons from odd and even trials of the same homogeneous (**b**) or heterogeneous condition (**c**).

**d-e** Proportions of global motion direction-selective PPC neurons from odd and even trials of the same homogeneous (**d**) or heterogeneous condition (**e**).


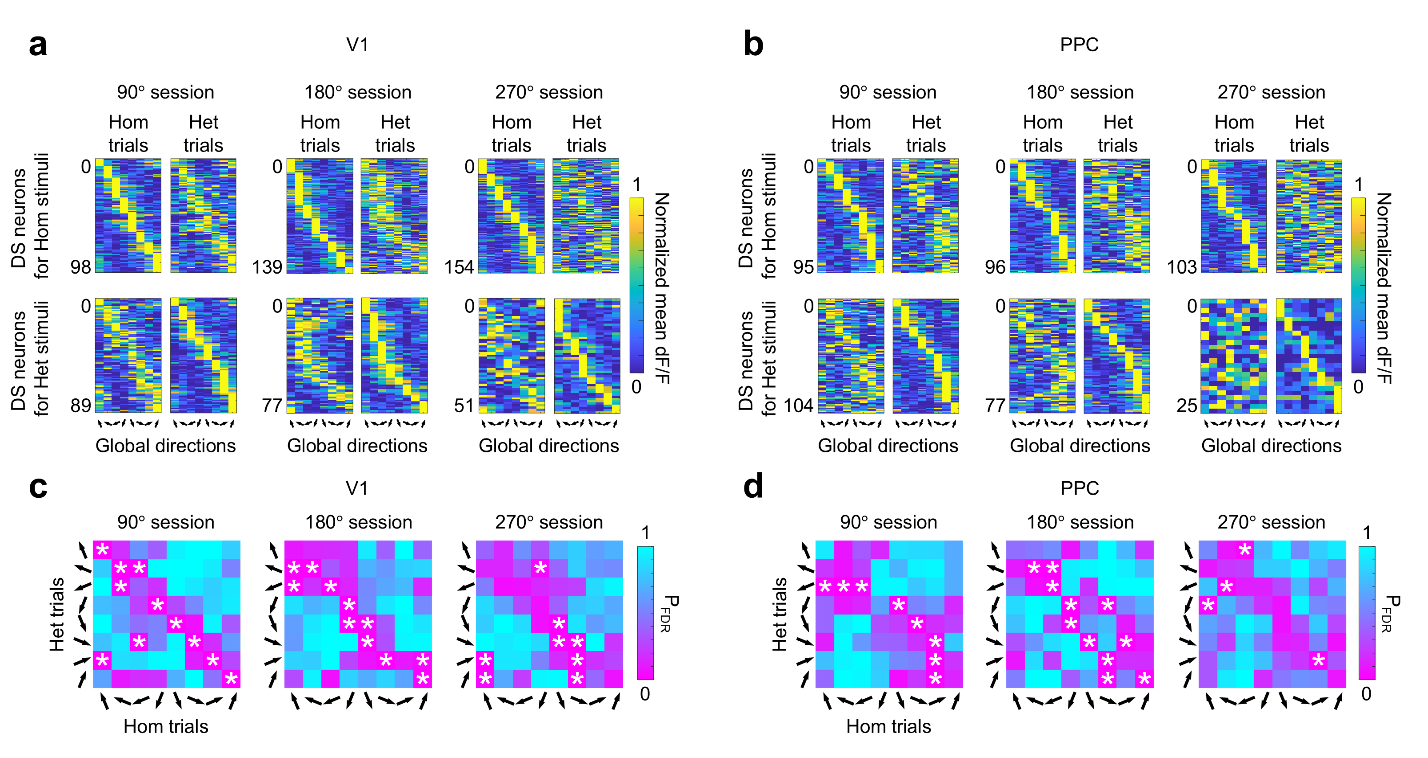


**Figure S7** Variability of global motion direction-selective V1 and PPC neurons during passive viewing

**a** The tuning curves of global motion direction-selective V1 neurons in 90°, 180°, and 270° sessions.

**b** The tuning curves of global motion direction-selective PPC neurons in 90°, 180°, and 270° sessions.

**c** The pairs of global motion directions of homogeneous and heterogeneous RDKs that elicited the maximal responses from all recorded V1 neurons in 90°, 180°, and 270° sessions.

**d** The pairs of global motion directions of homogeneous and heterogeneous RDKs that elicited the maximal responses from all recorded PPC neurons in 90°, 180°, and 270° sessions.

Asterisk indicates the proportion of neurons that is significantly larger than the surrogate proportion of neurons obtained from randomly shuffling trials (5,000 permutations, *p* < 0.05 based on FDR). Total numbers of V1 neurons are 424, 674, and 523 and total numbers of PPC neurons are 1050, 1123, and 1006 for the 90°, 180°, and 270° sessions, respectively.


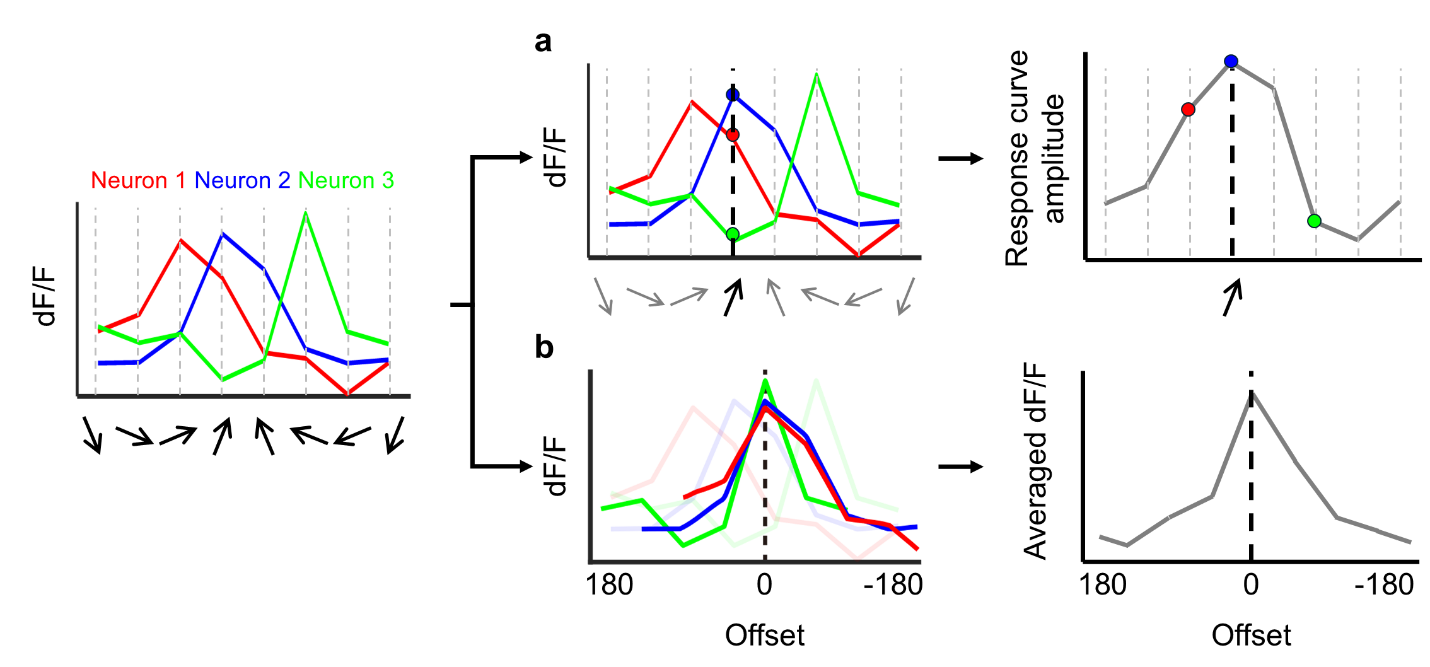


**Figure S8** Schematics of constructing population response curves and population-averaged tuning curves.

**a** Population response curve (PRC) constructed from *dF/F* responses of individual neurons to the given global motion direction (related to Figure 4).

**b** Zero-centered population-averaged tuning curve (related to Figure 2d).


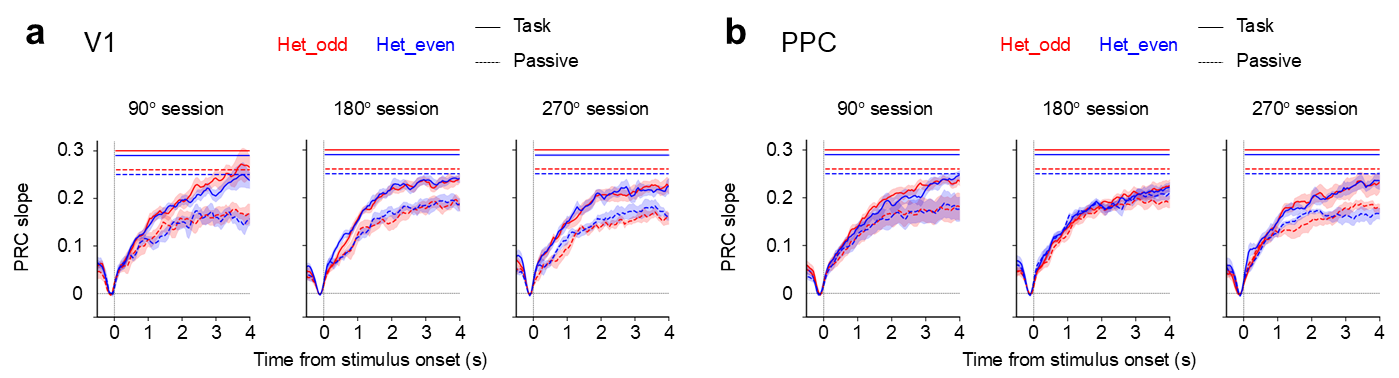


**Figure S9** **a**, **b** PRCs for heterogeneous trials split into odd and even subsets in V1 (a) and PPC (b) across the 90°, 180°, and 270° sessions. The encoding accuracies of the global motion directions are sharply increased after the stimulus onset. Red and blue color indicate homogeneous and heterogeneous trials, respectively. Shaded areas indicate ±SEM. Solid and dashed lines above the figures indicate the time-period when the PRC slope is significantly greater than 0 (p < 0.05, based on cluster extent) during motion categorization task and passive viewing, respectively. All PRC slopes were significant from 500ms to 4000ms.

Data presented as the mean ± SEM, V1 task N = 10, V1 passive N = 6, PPC task N = 7, PPC passive N = 8, statistical analysis by cluster-based permutation test (two-tailed; cluster-forming threshold p < 0.05, uncorrected; cluster-mass statistic; 5,000 permutations; FWER α = 0.05). Significant clusters are indicated by bars above the trace.


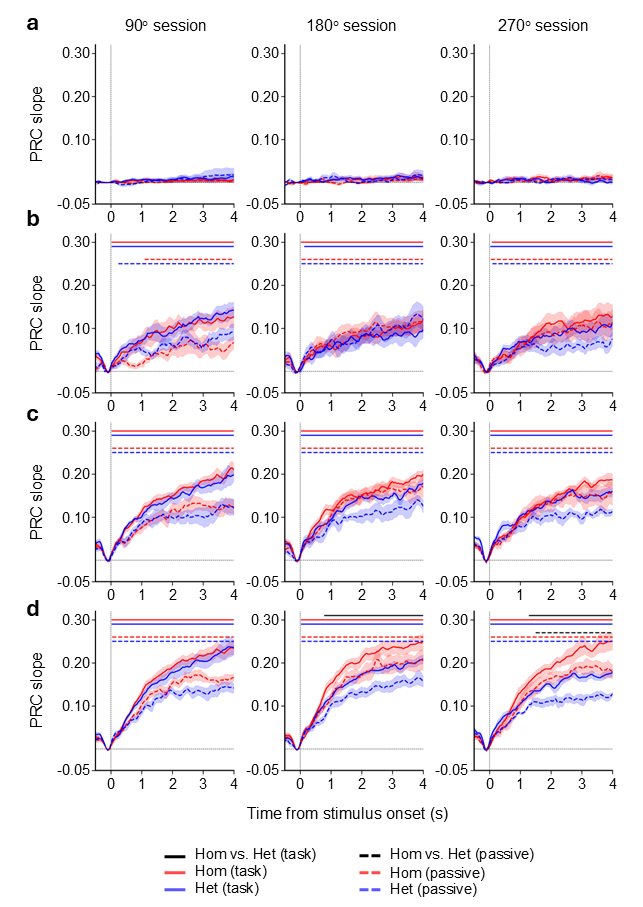


**Figure S10** PRCs in V1 calculated using different proportions of neurons in the 90°, 180°, and 270° sessions using the bottom 10% (**a**), 50% (**b**), 95% (**c**) and 100% (**d**) of all recorded neurons that are rank-ordered by their single-neuron tuning curve slopes tested against slopes of the trial-shuffled dataset. Red and blue color indicate homogeneous and heterogeneous trials, respectively. Error bars indicate ±SEM. Solid and dashed lines indicate the PRC slope during motion categorization task and passive viewing, respectively. **d** is identical to Figure 4d.

Data presented as the mean ± SEM, V1 task N = 10, V1 passive N = 6, PPC task N = 7, PPC passive N = 8, statistical analysis by cluster-based permutation test (two-tailed; cluster-forming threshold p < 0.05, uncorrected; cluster-mass statistic; 5,000 permutations; FWER α = 0.05). Significant clusters are indicated by bars above the trace.


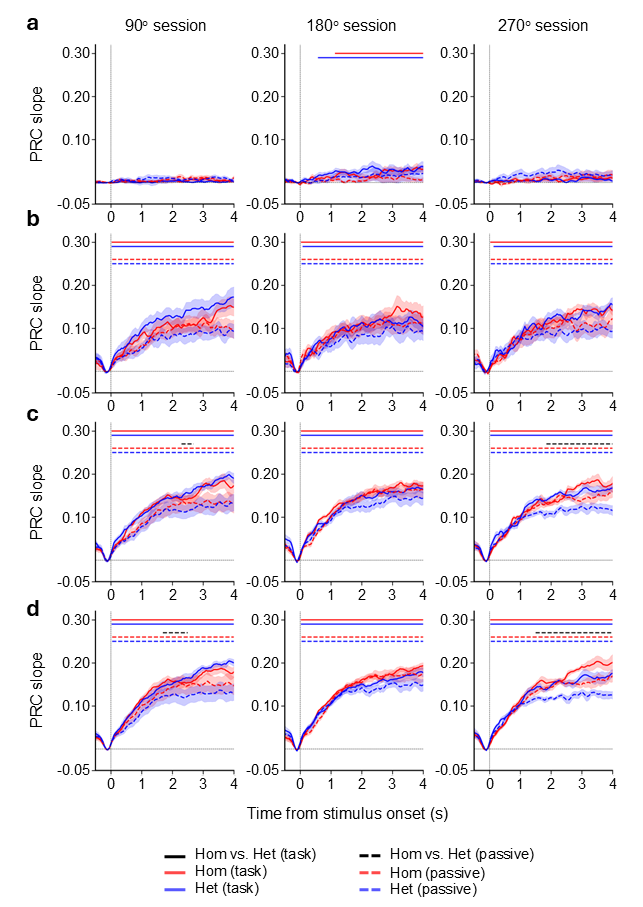


**Figure S5** PRCs in PPC calculated using different proportions of neurons in the 90°, 180°, and 270° sessions using the bottom 10% (**a**), 50% (**b**), 95% (**c**) and 100% (**d**) of all recorded neurons that are rank-ordered by their single-neuron tuning curve slopes tested against slopes of the trial-shuffled dataset. Red and blue color indicate homogeneous and heterogeneous trials, respectively. Error bars indicate ±SEM. Solid and dashed lines indicate the PRC slope during motion categorization task and passive viewing, respectively. **d** is identical to Figure 4e.

Data presented as the mean ± SEM, V1 task N = 10, V1 passive N = 6, PPC task N = 7, PPC passive N = 8, statistical analysis by cluster-based permutation test (two-tailed; cluster-forming threshold p < 0.05, uncorrected; cluster-mass statistic; 5,000 permutations; FWER α = 0.05). Significant clusters are indicated by bars above the trace.


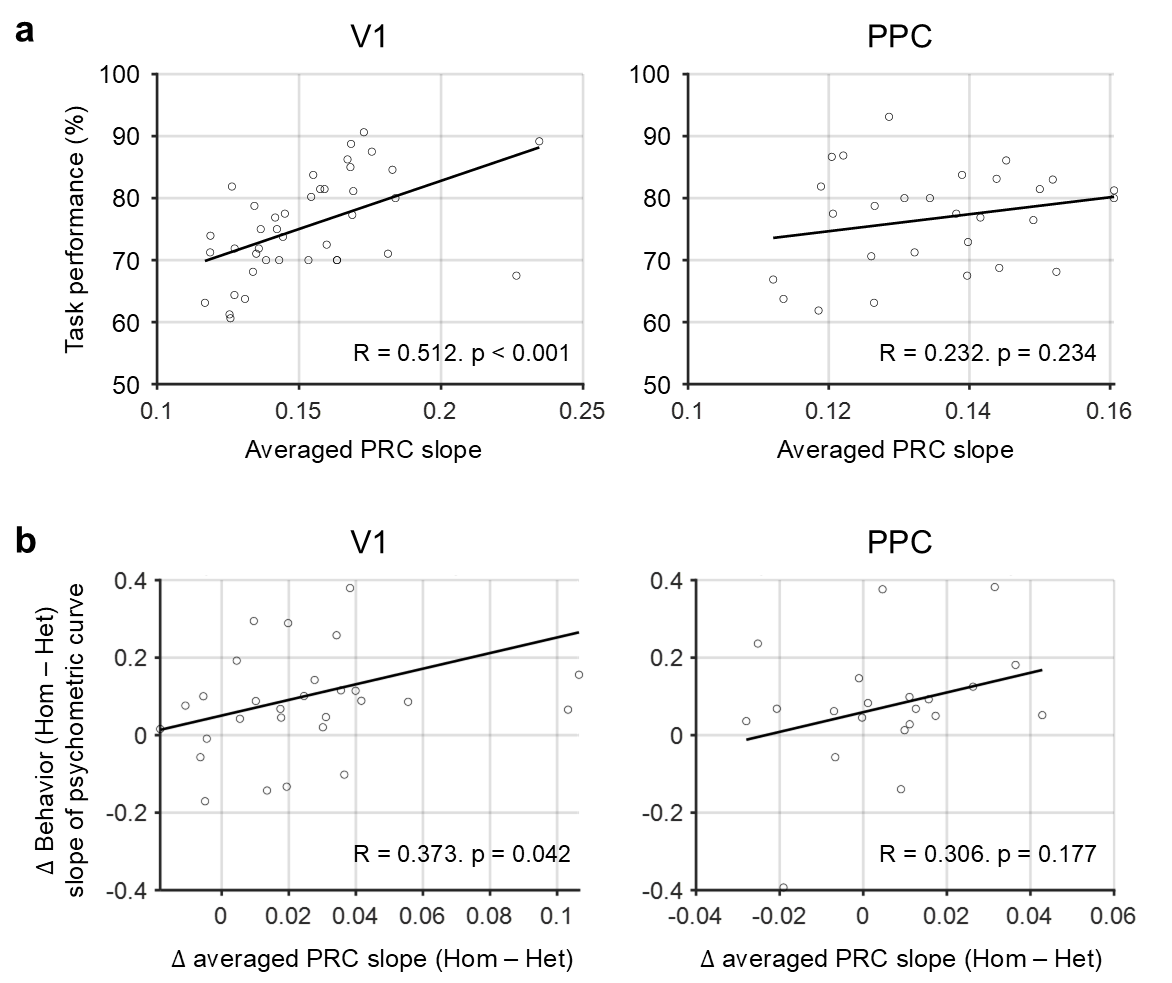


**Figure S12** Correlation between mean direction encoding accuracy (PRC slope) and category discrimination performance.

**a** Correlation between PRC slope averaged over the period of 0 – 4 s after stimulus onset (x-axis) and category discrimination accuracy (y-axis). V1 showed a significant positive correlation (r = 0.51, p = 0.00074), whereas PPC did not show a positive correlation (r = 0.23, p = 0.23). Black lines indicate linear regression lines.

**b** Correlation between the difference in PRC slope averaged over the period of 0 – 4 s after stimulus onset (x-axis) and the corresponding difference in psychometric curve slope between homogeneous and heterogeneous trials (y-axis). V1 showed a significant positive correlation (r = 0.37, p = 0.04), whereas PPC did not show a positive correlation (r = 0.31, p = 0.18). Black lines indicate linear regression lines.

Correlation was assessed with Pearson’s correlation (two-tailed).


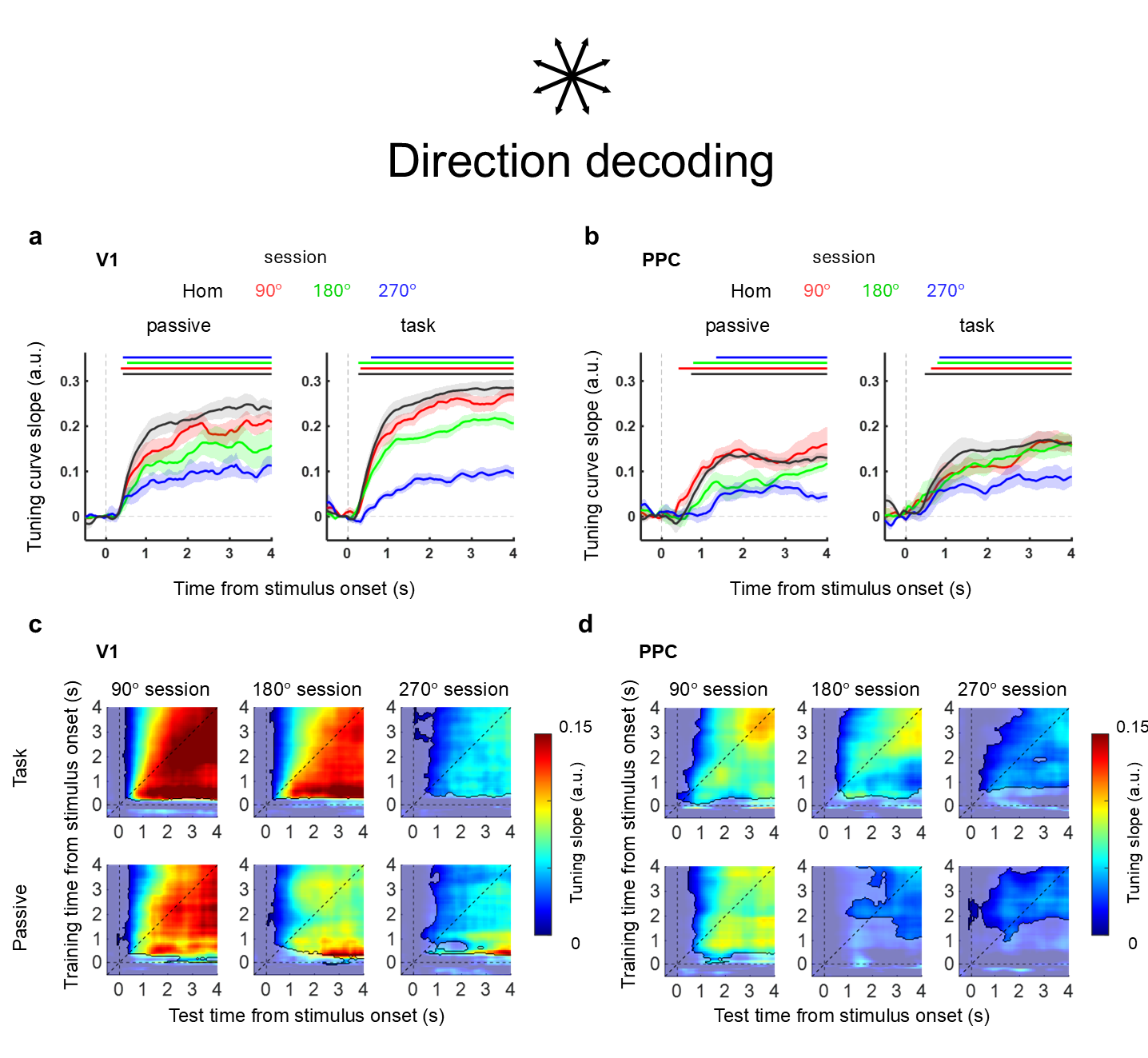


**Figure S6** **a, b** IEM-based reconstruction of population tuning curve of global mean motion direction in V1 (a) and PPC (b). Tuning curve slope was computed to summarize the reconstructed tuning curve at each time point (See details in the Method section). Black lines denote the tuning curve slope of homogeneous RDK as a function of time and red/green/blue lines denote the tuning curve slopes of the 90°/180°/270° RDKs as a function of time, respectively. At each time-point, IEM trained on homogeneous trials was tested on heterogeneous trials to reconstruct global motion direction information from heterogeneous trials. Leave-one-out cross-validation was used to reconstruct global motion direction information from homogeneous trials. Significant time points where tuning curve slope was significantly greater than 0 are marked by lines over the graphs based on cluster-based permutation tests (p < 0.05).

In V1 passive condition, significant clusters were: homogeneous session (black): 424-4000ms (p = 0.016); 90° session (red): 374–4000ms (p = 0.018); 180° session (green): 525-4000ms (p = 0.018); 270° session (blue): 424–4000ms (p = 0.013).

In V1 task condition, significant clusters were: homogeneous session (black): 273-4000ms (p < 0.001); 90° session (red): 323–4000ms (p < 0.001); 180° session (green): 273-4000ms (p < 0.001); 270° session (blue): 576–4000ms (p < 0.001).

In PPC passive condition, significant clusters were: homogeneous session (black): 727-4000ms (p = 0.005); 90° session (red): 424–4000ms (p = 0.003); 180° session (green): 778-4000ms (p = 0.005); 270° session (blue): 1333–4000ms (p = 0.004).

In PPC task condition, significant clusters were: homogeneous session (black): 475-4000ms (p = 0.013); 90° session (red): 626–4000ms (p = 0.008); 180° session (green): 778-4000ms (p = 0.008); 270° session (blue): 828–4000ms (p = 0.013).

**c, d** Temporal generalization (TG) maps of IEM-based reconstruction of global mean motion direction in V1 (c) and PPC (d). For each region, three TG maps are shown left to right for the 90°, 180°, and 270° sessions. At each training time point, IEM trained on homogeneous trials was tested on heterogeneous trials across all test time points to reconstruct global motion direction from heterogeneous trials. Axes indicate training time (y) and testing time (x) relative to stimulus onset; vertical and horizontal dotted lines mark stimulus onset. Significant clusters are outlined (cluster-based permutation test, p < 0.05).

Data presented as the mean ± SEM, V1 task N = 10, V1 passive N = 6, PPC task N = 7, PPC passive N = 8, statistical analysis by cluster-based permutation test (two-tailed; cluster-forming threshold p < 0.05, uncorrected; cluster-mass statistic; 5,000 permutations; FWER α = 0.05). Significant clusters are indicated by bars above the trace (a, b) and outlined (c, d).


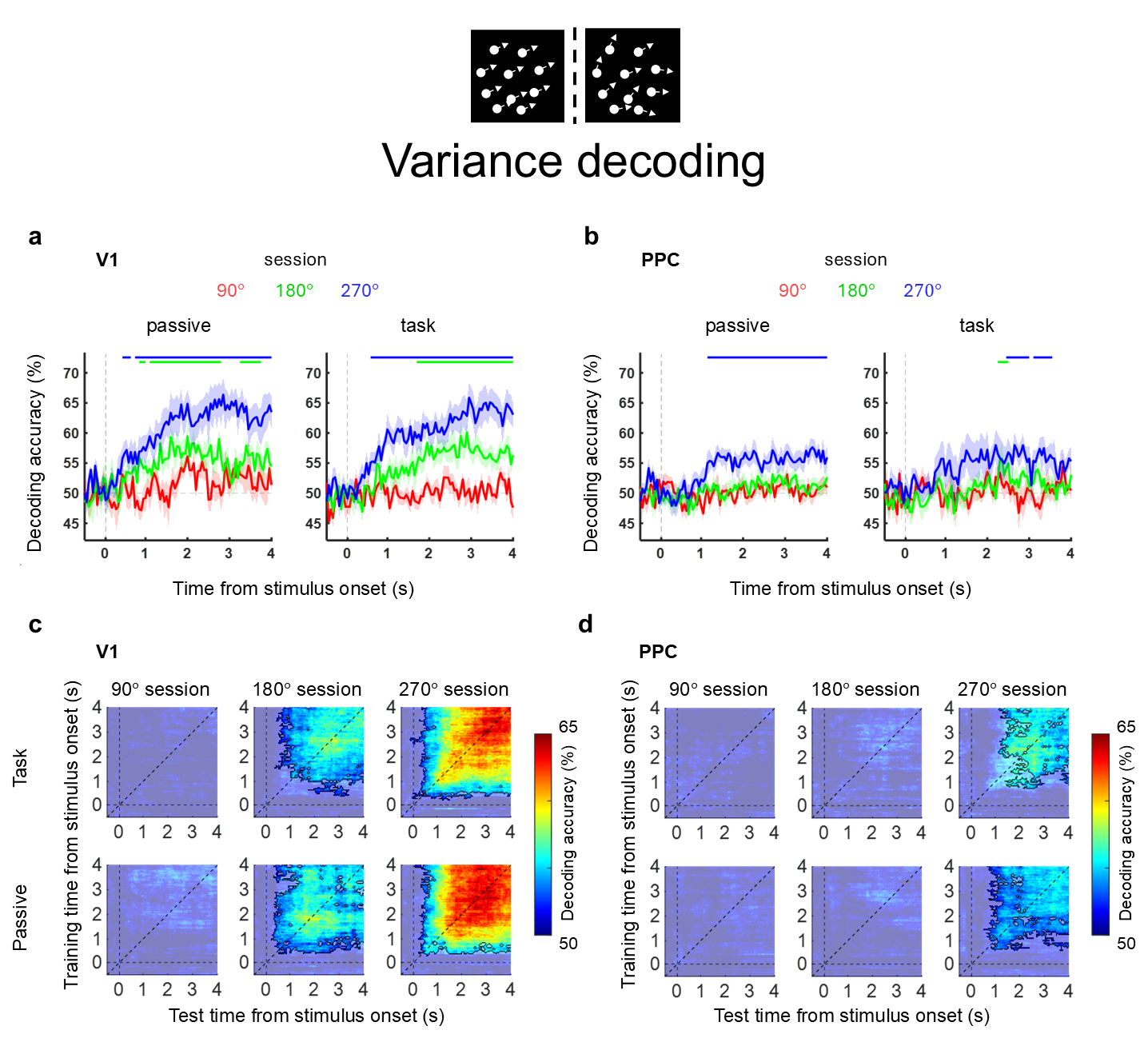


**Figure S7** **a, b** Time-resolved variance decoding using SVM (leave-one-out cross-validation) in V1 (a) and PPC (b). Red, green and blue lines denote decoding accuracies from the 90°, 180° and 270° sessions, respectively. At each time point, SVM classifiers were trained and tested to distinguish homogeneous from heterogeneous trials in V1 and PPC, respectively. Time periods where the decoding accuracy was significantly greater than chance level are marked by lines over the graphs based on a cluster-based permutation test (p < 0.05).

In V1 passive condition, significant time clusters were: 180° session (green): 828–980ms (p = 0.026), 1081-2798ms (p = 0.011) and 3252-3757ms (p = 0.026); 270° session (blue): 424-626ms (p = 0.048) and 727–4000ms (p = 0.017).

In V1 task condition, significant time clusters were: 180° session (green): 1687–4000ms (p = 0.002); 270° session (blue): 576–4000ms (p = 0.002).

In PPC passive condition, significant time clusters were: 270° session (blue): 1131-4000ms (p = 0.004).

In PPC task condition, significant time clusters were: 180° session (green): 2242-2495ms (p = 0.016); 270° session (blue): 2444-3000ms (p = 0.028) and 3101-3555 (p = 0.028).

**c, d** Temporal generalization (TG) maps of time-generalized variance decoding in V1 (c) and PPC (d). For each region, three TP maps are shown from left to right for the 90°, 180°, and 270° sessions. At each training time point, SVM classifiers trained to discriminate homogeneous from heterogeneous trials were tested across all time points (leave-one-out cross-validation). Axes indicate training time (y) and testing time (x) relative to stimulus onset; vertical and horizontal dotted lines mark stimulus onset. Significant clusters (>50%) are outlined (cluster-based permutation test, p < 0.05).

Data presented as the mean ± SEM, V1 task N = 10, V1 passive N = 6, PPC task N = 7, PPC passive N = 8, statistical analysis by cluster-based permutation test (two-tailed; cluster-forming threshold p < 0.05, uncorrected; cluster-mass statistic; 5,000 permutations; FWER α = 0.05). Significant clusters are indicated by bars above the trace (a, b) and outlined (c, d).


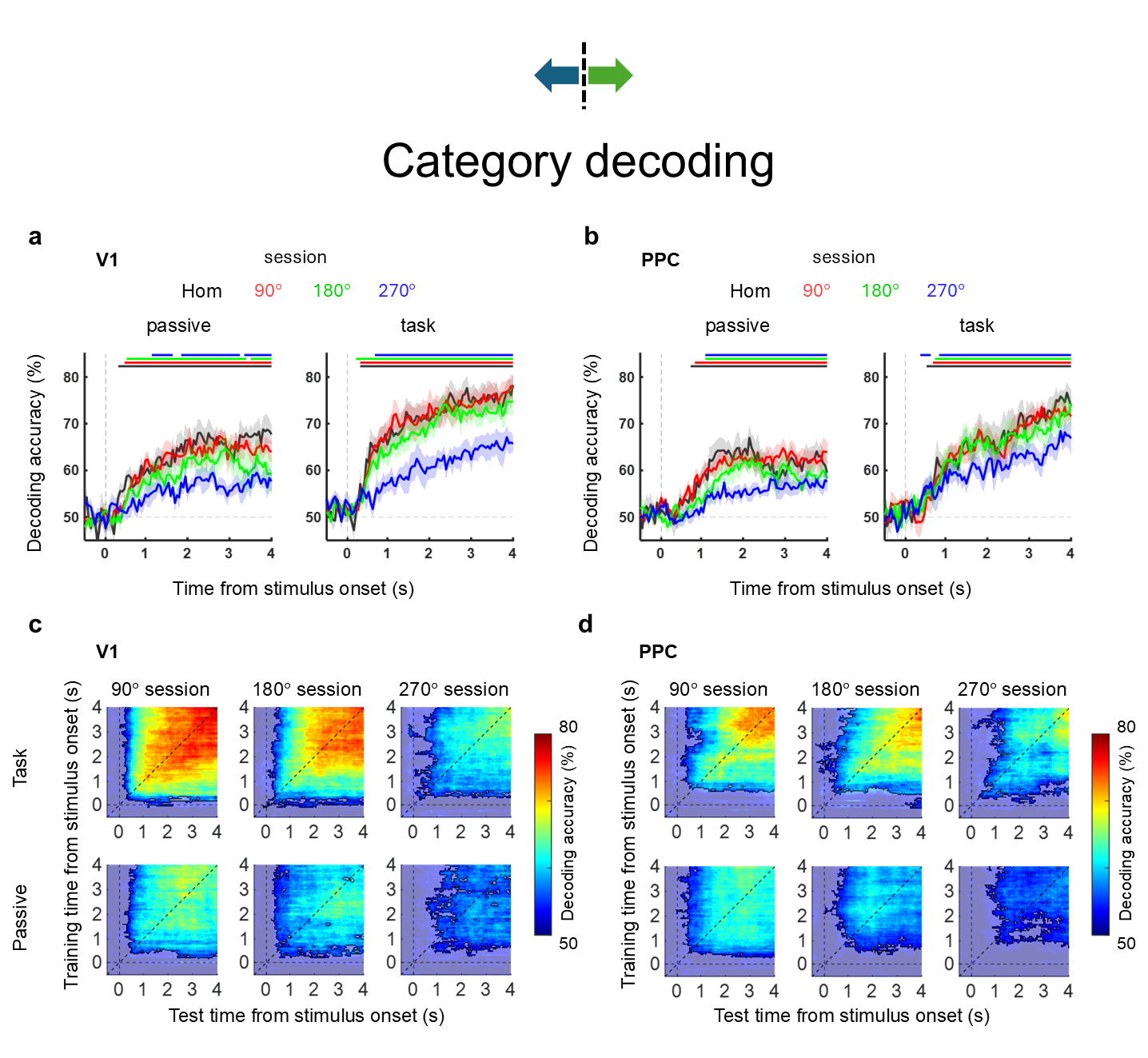


**Figure S15** **a, b** Time-resolved category decoding using SVM in V1 (a) and PPC (b). Black lines denote the classification accuracy of left vs right category for the homogeneous session and red/green/blue lines denote classification accuracy of left vs right category for the 90°/180°/270° session, respectively. At each time-point, a binary SVM trained on homogeneous trials to classify left vs. right was tested on heterogeneous trials. Also leave-one-out cross-validation procedure was used to compute the decoding accuracy for homogeneous trials. Significant time points where the left and right categories were classified above the chance level are indicated by lines over the graphs based on cluster-based permutation tests (p < 0.05).

In V1 passive condition, significant clusters were: homogeneous session (black): 323-4000ms (p = 0.019); 90° session (red): 475–4000ms (p = 0.012); 180° session (green): 525-3404ms (p = 0.043) and 3505-4000ms (p = 0.043); 270° session (blue): 1131–1636ms (p = 0.035), 1838–3252ms (p = 0.035) and 3353–4000ms (p = 0.035).

In V1 task condition, significant clusters were: homogeneous session (black): 323-4000ms (p = 0.002); 90° session (red): 323–4000ms (p = 0.0001); 180° session (green): 222-4000ms (p = 0.0001); 270° session (blue): 677–4000ms (p = 0.002).

In PPC passive condition, significant clusters were: homogeneous session (black): 727-4000ms (p = 0.004); 90° session (red): 828–4000ms (p = 0.005); 180° session (green): 1081-4000ms (p = 0.005); 270° session (blue): 1081–4000ms (p = 0.008).

In PPC task condition, significant clusters were: homogeneous session (black): 525-4000ms (p = 0.011); 90° session (red): 677–4000ms (p = 0.01); 180° session (green): 727-4000ms (p = 0.007); 270° session (blue): 374–626ms (p = 0.043) and 828–4000ms (p = 0.006).

**c, d** Temporal generalization (TG) maps of category decoding using SVM in V1 (c) and PPC (d). For each region, three TG maps are shown from left to right for the 90°, 180°, and 270° sessions. At each training time point, a binary SVM trained on homogeneous trials to classify left vs. right was tested on heterogeneous trials across all time points. Axes indicate training time (y) and testing time (x) relative to stimulus onset; vertical and horizontal dotted lines mark stimulus onset. Significant clusters (>50%) are outlined (cluster-based permutation test, p < 0.05).

Data presented as the mean ± SEM, V1 task N = 10, V1 passive N = 6, PPC task N = 7, PPC passive N = 8, statistical analysis by cluster-based permutation test (two-tailed; cluster-forming threshold p < 0.05, uncorrected; cluster-mass statistic; 5,000 permutations; FWER α = 0.05). Significant clusters are indicated by bars above the trace (a, b) and outlined (c, d).

**Table S1.** Statistical comparison of behavioral performance between easy and hard (related to Figure 1d)

| **Title** | **Figure** | **Comparison** | **Statistical Test** | **Test Statistic (df)** | **p-value** | **Correction Method** | **Significance** |
| --- | --- | --- | --- | --- | --- | --- | --- |
| **Behavioral performance difference between easy and hard direction** | x | Hom Easy vs Hard (90° session) | Paired t-test | t(16) = 15.33 | < 1 × 10⁻¹⁰ | None | o |
|  | x | Hom Easy vs Hard (180° session) | Paired t-test | t(16) = 17.6 | < 1 × 10⁻¹¹ | None | o |
|  | x | Hom Easy vs Hard (270° session) | Paired t-test | t(16) = 17.6 | < 1 × 10⁻¹¹ | None | o |
|  | x | Het Easy vs Hard (90° session) | Paired t-test | t(16) = 15.55 | < 1 × 10⁻¹⁰ | None | o |
|  | x | Het Easy vs Hard (180° session) | Paired t-test | t(16) = 9.07 | < 1 × 10⁻⁷ | None | o |
|  | x | Het Easy vs Hard (270° session) | Paired t-test | t(16) = 9.07 | < 1 × 10⁻⁷ | None | o |

**Table S2.** Chi-square tests assessing if the observed proportion of global motion direction-selective neurons in both Hom and Het conditions is different from the expected proportion that global motion direction-selective neurons in each of Hom and Het condition would show direction-selectivity in both conditions by chance (related to Figure 2c and S1b).

| **Title** | **Figure** | **Region** | **Comparison** | **Statistical Test** | **Test Statistic (df)** | **p-value** | **Correction Method** | **Significance** |
| --- | --- | --- | --- | --- | --- | --- | --- | --- |
| **The significance of the proportion of global motion DS neurons under both Hom and Het conditions** | Fig. 2c | V1 | Hom and Het (90° session) | Chi-square test | χ²(3, N = 1312) = 1304.04 | < 0.001 | None | o |
|  | Fig. 2c | V1 | Hom and Het (180° session) | Chi-square test | χ²(3, N = 1407) = 1399.03 | < 0.001 | None | o |
|  | Fig. 2c | V1 | Hom and Het (270° session) | Chi-square test | χ²(3, N = 1367) = 1359.03 | < 0.001 | None | o |
|  | Fig. S2b | PPC | Hom and Het (90° session) | Chi-square test | χ²(3, N = 997) = 989.09 | < 0.001 | None | o |
|  | Fig. S2b | PPC | Hom and Het (180° session) | Chi-square test | χ²(3, N = 984) = 976.09 | < 0.001 | None | o |
|  | Fig. S2b | PPC | Hom and Het (270° session) | Chi-square test | χ²(3, N = 996) = 988.09 | < 0.001 | None | o |

**Table S3.** Results of cluster-based permutation tests in Figure 4d and Figure 4e

| **Figure** | **conditions** | **Significant time points (sec)** |
| --- | --- | --- |
| Fig 4d (V1) | 90 session (Homogeneous, Task) vs 0 | 0.05 to 4 |
|  | 90 session (Heterogeneous, Task) vs 0 | 0.05 to 4 |
|  | 90 session (Homogeneous, Passive) vs 0 | 0.05 to 4 |
|  | 90 session (Heterogeneous, Passive) vs 0 | 0.05 to 4 |
|  |  |  |
|  | 180 session (Homogeneous, Task) vs 0 | 0.05 to 4 |
|  | 180 session (Heterogeneous, Task) vs 0 | 0.05 to 4 |
|  | 180 session (Homogeneous, Passive) vs 0 | 0.05 to 4 |
|  | 180 session (Heterogeneous, Passive) vs 0 | 0.05 to 4 |
|  | 180 session (Homogeneous, Task) vs (Heterogeneous, Task) | 0.8 to 4 |
|  |  |  |
|  | 180 session (Homogeneous, Task) vs 0 | 0.05 to 4 |
|  | 180 session (Heterogeneous, Task) vs 0 | 0.05 to 4 |
|  | 180 session (Homogeneous, Passive) vs 0 | 0.05 to 4 |
|  | 180 session (Heterogeneous, Passive) vs 0 | 0.05 to 4 |
|  | 180 session (Homogeneous, Task) vs (Heterogeneous, Task) | 1.3 to 4 |
|  | 180 session (Homogeneous, Passive) vs (Heterogeneous, Passive) | 1.5 to 4 |
| Fig 4e (PPC) | 90 session (Homogeneous, Task) vs 0 | 0.05 to 4 |
|  | 90 session (Heterogeneous, Task) vs 0 | 0.05 to 4 |
|  | 90 session (Homogeneous, Passive) vs 0 | 0.05 to 4 |
|  | 90 session (Heterogeneous, Passive) vs 0 | 0.05 to 4 |
|  | 90 session (Homogeneous, Passive) vs (Heterogeneous, Passive) | 1.7 to 2.5 |
|  |  |  |
|  | 180 session (Homogeneous, Task) vs 0 | 0.05 to 4 |
|  | 180 session (Heterogeneous, Task) vs 0 | 0.05 to 4 |
|  | 180 session (Homogeneous, Passive) vs 0 | 0.05 to 4 |
|  | 180 session (Heterogeneous, Passive) vs 0 | 0.05 to 4 |
|  |  |  |
|  | 180 session (Homogeneous, Task) vs 0 | 0.05 to 4 |
|  | 180 session (Heterogeneous, Task) vs 0 | 0.05 to 4 |
|  | 180 session (Homogeneous, Passive) vs 0 | 0.05 to 4 |
|  | 180 session (Heterogeneous, Passive) vs 0 | 0.05 to 4 |
|  | 180 session (Homogeneous, Passive) vs (Heterogeneous, Passive) | 1.5 to 4 |

**Table S4.** Results of statistical tests in Figure 5b.

| **Title** | **Figure** | **Comparison** | **Statistical Test** | **Test Statistic (df)** | **p-value** | **Correction Method** | **Significance** |
| --- | --- | --- | --- | --- | --- | --- | --- |
| **Bias (outliers > 1.5 IQR removed)** | Fig 5 | Hom Passive bias vs 0 | Paired t-test | t(13)=1.47 | 0.08265782 | FDR | x |
|  | Fig 5 | Hom Task bias vs 0 | Paired t-test | t(15)=5.23 | 0.00010262 | FDR | o |
|  | Fig 5 | Het 90 Passive bias vs 0 | Paired t-test | t(5)=0.85 | 0.3767806 | FDR | x |
|  | Fig 5 | Het 90 Task bias vs 0 | Paired t-test | t(9)=5.28 | 0.00152533 | FDR | o |
|  | Fig 5 | Het 180 Passive bias vs 0 | Paired t-test | t(4)=-3.57 | 0.98832094 | FDR | x |
|  | Fig 5 | Het 180 Task bias vs 0 | Paired t-test | t(6)=3.17 | 0.02895781 | FDR | o |
|  | Fig 5 | Het 270 Passive bias vs 0 | Paired t-test | t(4)=0.74 | 0.3767806 | FDR | x |
|  | Fig 5 | Het 270 Task bias vs 0 | Paired t-test | t(9)=0.20 | 0.50815096 | FDR | X |

**Table S5.** Results of cluster-based permutation tests in Figure 6b and Figure 6c

| **Figure** | **conditions** | **Significant time points 1** | **Significant time points 2** | **Significant time points 3** | **Significant time points 4** |
| --- | --- | --- | --- | --- | --- |
| Fig 6b | V1 task 90 VAR | none |  |  |  |
|  | V1 task 90 CAT | none |  |  |  |
|  | V1 task 90 DIR | 0.35 to 4 |  |  |  |
|  |  |  |  |  |  |
|  | V1 task 180 VAR | 0.65 to 1 | 1.4 to 1.8 | 2.05 to 2.75 | 3.05 to 3.8 |
|  | V1 task 180 CAT | none |  |  |  |
|  | V1 task 180 DIR | 0.2 to 4 |  |  |  |
|  |  |  |  |  |  |
|  | V1 task 270 VAR | 1 to 4 |  |  |  |
|  | V1 task 270 CAT | none |  |  |  |
|  | V1 task 270 DIR | 0.95 to 4 |  |  |  |
|  |  |  |  |  |  |
|  | V1 passive 90 VAR | none |  |  |  |
|  | V1 passive 90 CAT | none |  |  |  |
|  | V1 passive 90 DIR | 0.35 to 4 |  |  |  |
|  |  |  |  |  |  |
|  | V1 passive 180 VAR | 1.5 to 2.2 | 2.9 to 3.2 | 3.35 to 3.9 |  |
|  | V1 passive 180 CAT | none |  |  |  |
|  | V1 passive 180 DIR | 0.25 to 4 |  |  |  |
|  |  |  |  |  |  |
|  | V1 passive 270 VAR | 0.65 to 4 |  |  |  |
|  | V1 passive 270 CAT | none |  |  |  |
|  | V1 passive 270 DIR | 0.35 to 0.9 | 1 to 4 |  |  |
| Fig 6c | PPC task 90 VAR | none |  |  |  |
|  | PPC task 90 CAT | 2.5 to 4 |  |  |  |
|  | PPC task 90 DIR | 2.65 to 4 |  |  |  |
|  |  |  |  |  |  |
|  | PPC task 180 VAR | none |  |  |  |
|  | PPC task 180 CAT | 1.05 to 4 |  |  |  |
|  | PPC task 180 DIR | none |  |  |  |
|  |  |  |  |  |  |
|  | PPC task 270 VAR | none |  |  |  |
|  | PPC task 270 CAT | 1.7 to 4 |  |  |  |
|  | PPC task 270 DIR | none |  |  |  |
|  |  |  |  |  |  |
|  | PPC passive 90 VAR | none |  |  |  |
|  | PPC passive 90 CAT | 0.75 to 2.3 |  |  |  |
|  | PPC passive 90 DIR | 0.9 to 2.9 | 3.2 to 4 |  |  |
|  |  |  |  |  |  |
|  | PPC passive 180 VAR | none |  |  |  |
|  | PPC passive 180 CAT | 1.55 to 3.85 |  |  |  |
|  | PPC passive 180 DIR | 1 to 2.15 | 2.25 to 3.5 |  |  |
|  |  |  |  |  |  |
|  | PPC passive 180 VAR | 2.95 to 4 |  |  |  |
|  | PPC passive 180 CAT | 2.6 to 4 |  |  |  |
|  | PPC passive 180 DIR | none |  |  |  |

**Table S6.** Results of cluster-based permutation tests in Figure 6e and Figure 6f

| **Figure** | **conditions** | **Significant time points 1 (s)** | **Significant time points 2 (s)** | **Significant time points 3 (s)** |
| --- | --- | --- | --- | --- |
| Fig 6e | V1 passive WCD | 0.2 to 4 |  |  |
|  | V1 passive BCD | 0.5 to 4 |  |  |
|  |  |  |  |  |
|  | V1 task WCD | 0.4 to 4 |  |  |
|  | V1 task BCD | 0.4 to 4 |  |  |
| Fig 6f | PPC passive WCD | 0.9 to 2.6 | 2.7 to 4 |  |
|  | PPC passive BCD | 0.9 to 4 |  |  |
|  | PPC passive BCD vs WCD | 2.6 to 3.6 |  |  |
|  |  |  |  |  |
|  | PPC task WCD | 0.7 to 4 |  |  |
|  | PPC task BCD | 0.7 to 4 |  |  |
|  | PPC task BCD vs WCD | 0.7 to 1.2 | 1.4 to 1.9 | 2.5 to 4 |

**Movie S1. Example RDKs in 90° session**

**Movie S2. Example RDKs in 180° session**

**Movie S3. Example RDKs in 270° session**
